# Generative and reinforcement learning approaches for the automated de novo design of bioactive compounds

**DOI:** 10.1038/s42004-022-00733-0

**Published:** 2022-10-18

**Authors:** Maria Korshunova, Niles Huang, Stephen Capuzzi, Dmytro S. Radchenko, Olena Savych, Yuriy S. Moroz, Carrow I. Wells, Timothy M. Willson, Alexander Tropsha, Olexandr Isayev

**Affiliations:** 1 Department of Chemistry, Mellon College of Science, Carnegie Mellon University, Pittsburgh, PA USA; 2 Computational Biology Department, School of Computer Science, Carnegie Mellon University, Pittsburgh, PA USA; 3 Department of Biochemistry, University of Oxford, Oxford, UK; 4 Laboratory for Molecular Modeling, UNC Eshelman School of Pharmacy, University of North Carolina at Chapel Hill, Chapel Hill, NC USA; 5 Enamine Ltd, 78 Chervonotkatska Street, Kyiv, 02094 Ukraine; 6 Taras Shevchenko National University of Kyiv, Volodymyrska Street 60, Kyiv, 01601 Ukraine; 7 Chemspace LLC, Chervonotkatska Street 85, Suite 1, Kyiv, 02094 Ukraine; 8 Structual Genomics Consortium, UNC Eshelman School of Pharmacy, University of North Carolina at Chapel Hill, Chapel Hill, NC USA

**Keywords:** Cheminformatics, Small molecules

## Abstract

Deep generative neural networks have been used increasingly in computational chemistry for *de novo* design of molecules with desired properties. Many deep learning approaches employ reinforcement learning for optimizing the target properties of the generated molecules. However, the success of this approach is often hampered by the problem of sparse rewards as the majority of the generated molecules are expectedly predicted as inactives. We propose several technical innovations to address this problem and improve the balance between exploration and exploitation modes in reinforcement learning. In a proof-of-concept study, we demonstrate the application of the deep generative recurrent neural network architecture enhanced by several proposed technical tricks to design inhibitors of the epidermal growth factor (EGFR) and further experimentally validate their potency. The proposed technical solutions are expected to substantially improve the success rate of finding novel bioactive compounds for specific biological targets using generative and reinforcement learning approaches.

## Abstract (web summary)

Deep generative neural networks are increasingly exploited for drug discovery, but often the majority of generated molecules are predicted to be inactive. Here, an optimized protocol for generative models with reinforcement learning is derived and applied to design potent epidermal growth factor inhibitors.

## Introduction

### Deep and reinforcement learning in drug discovery

The development and application of deep-generative models for de novo design of molecules with the desired properties have emerged as an important modern research direction in Computer-Assisted Drug Discovery (CADD)^1–4^. Deep-generative models can be categorized by the types of molecular representation employed in model development. The most commonly used representations are SMILES strings^5^ and molecular graphs. Multiple models for generating SMILES strings^6–9^ and molecular graphs^10–14^ corresponding to synthetically feasible novel molecules have been proposed. Initially, these models are typically trained on a diverse dataset of molecules so that they can generate a broad distribution of molecules. We shall denote a *naïve* generative model as a model that has been trained on a generic dataset prior to any specific property optimization.

Reinforcement learning (RL)^7,15,16^ has been a popular strategy for optimizing properties of the generated molecules. For example, Olivecrona et al.^6^ and Blaschke et al.^17^ proposed the REINVENT algorithm and memory-assisted reinforcement learning, respectively, and demonstrated how these approaches could maximize the predicted activity of generated molecules against the 5-hydroxytryptamine receptor type 1A (HTR1A) and the dopamine type 2 receptor (DRD2). Another recent example is the RationaleRL algorithm proposed by Jin et al.^18^. The authors used RationaleRL to maximize the predicted activity of inhibitors against glycogen synthase kinase-3 beta (GSK3β) and c-Jun N-terminal kinase-3 (JNK3). Born et al.^19^ proposed performing optimization with RL on merged protein/ligand latent spaced constructed by the VAE. Unfortunately, the aforementioned studies included no experimental validation of the proposed computational hits. Notably, Zhavoronkov et al.^20^ not only proposed a novel generative tensorial reinforcement-learning algorithm, but also used their method to design potent DDR1 kinase inhibitors, and performed experimental validation of virtual hits.

Most theoretical studies on de novo molecular design employ optimization tasks for properties LogP^21^ and Quantitative Estimate of Druglikeness (QED)^22^, or the benchmark collection proposed in GuacaMol^23^. Such tasks employ objective metrics obtained directly from a molecule’s SMILES^5^ or underlying molecular graph through a scoring function. These scoring functions return continuous values that can be used to assign a reward to generated molecules. For example, the Quantitative Estimate of Druglikeness score (QED) has values between 0 and 1.0, with 0 being least drug-like and 1.0 being most drug-like. In such a case, every generated molecule would receive a continuous score: the bigger score values will correspond to bigger reward values, and vice versa. Moreover, a naïve generative model pre-trained on a dataset of drug-like compounds such as ChEMBL^24^ would produce molecules with relatively high QED values (see Fig. S1). In this case, optimization of the generative model via reinforcement learning will proceed efficiently as every generated molecule would get a score. Indeed, the efficient optimization of the QED score has been demonstrated many times in the literature^10,21,25^. These benchmarks are unable to simulate tasks with *sparse rewards*, such as designing molecules with high activity against a specific protein target. In such a case, only a small fraction of generated molecules possess the target property, which leads to reward sparsity during model training.

### The problem of sparse rewards in reinforcement learning

In contrast to physical properties such as LogP that can be calculated directly from molecular structure, the biological activity of a novel compound designed to bind the desired protein target cannot be predicted from its chemical structure alone. A common way to predict the binding affinity of novel, untested ligands is by using Quantitative Structure-Activity Relationship (QSAR) models^26,27^ trained on historical experimental data for a protein target of interest using machine-learning techniques. These models have either continuous outputs (pKd, pIC50, etc.) for regression problems or categorical outputs (active/inactive class label in a binary case) for classification problems. QSAR models could, in principle, be used to construct a reward function for reinforcement learning to optimize the binding affinity of generated molecules, as was shown, for instance, in our previous publication^7^. However, unlike physical molecular properties like LogP that every molecule possesses, specific bioactivity is a target property that exists for only a small fraction of molecules, which leads to the reward *sparseness* in the generative models. This *sparse rewards* problem represents a serious obstacle for the effective use of reinforcement learning for designing molecules with high activity. Indeed, the low success probability often leads to the overwhelming majority of training trajectories resulting in a zero reward, which implies that the reinforcement-learning agent or policy network struggles to explore the environment and learn the optimal strategy for maximizing the expected reward^28–30^. Thus, a promising molecule with high bioactivity for a protein of interest is unlikely to be observed if molecules are randomly sampled from a naïve generative model.

Training the generative network to optimize the potency of generated molecules against a desired protein target is an excellent example of a reinforcement-learning problem with sparse rewards. There is a very low chance of observing a molecule with high potency when sampled randomly from the distribution of unoptimized generative model. During training procedure with RL, training examples are produced by the generative model. The model trained just on negative examples (molecules with low potency values) will unlikely discover positive examples (molecules with high potency values). In this study, we demonstrate that the naïve generative model produces molecules predicted to be inactive in most cases. Under such a scenario, the naïve generative model rarely observes good examples and fails to maximize the active class probability for generated ligands. We further address this problem by proposing a set of heuristic approaches (a “bag of tricks”) combined with reinforcement learning in the sparse rewards situation to increase the efficiently of optimizing the structures of generated molecules to have higher predicted active class probability. Using the epidermal growth factor receptor (EGFR) ligands as a case study, we show that by combining a reinforcement-learning pipeline for generative model optimization with proposed heuristics, we could overcome sparse reward issues and successfully rediscover known active scaffolds for EGFR using the feedback from the classification QSAR model only. In addition to methodological advances, we also performed experimental bioassay validation of the novel generated hit molecules, which confirmed the experimental activity of virtual hits.

### Major findings

We performed a series of experiments that resulted in the following chief observations:

1. The generative model trained with only the policy gradient algorithm could not discover any molecules with high active class probability for EGFR due to sparse rewards.
2. The combination of policy gradient algorithm with proposed fine-tuning by (i) transfer learning, (ii) experience replay, and (iii) real-time reward shaping resulted in much better exploration and an increased number of generated molecules with high active class probabilities.
3. Experimental testing of selected computational hits that could be obtained from a commercial source validated the efficiency of our proposed approach for discovering novel bioactive molecules.

Below, we discuss how we arrived at the above observations. Overall, the section consists of two main parts. In the first part, we describe our computational analysis concerning the first two observations. In the second part, we discuss the generation, selection, and experimental bioactivity testing of computational hit compounds for an important cancer biological target, epidermal growth factor receptor (EGFR). The most active compound featured a privileged EGFR scaffold found in the known active molecules. Notably, the training set was not enriched for this scaffold as compared to other scaffolds and this scaffold was not used selectively as part of the reinforcement-learning procedure.

## Results and discussion

### Model pipeline

**Fig. 1:**
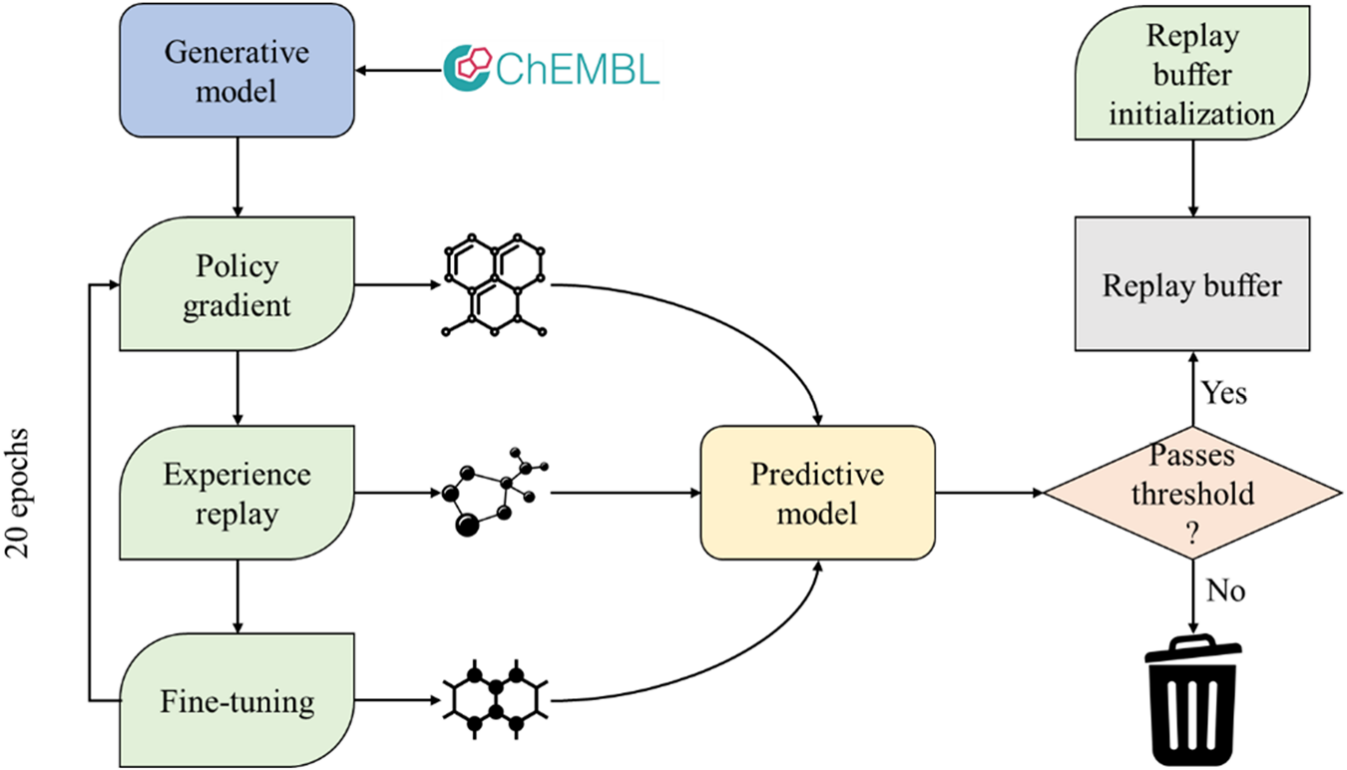
Pipeline of model training. The model was pre-trained on ChEMBL data and then trained for 20 epochs. Each epoch consists of three steps: policy gradient, policy experience replay, and fine-tuning. At the end of each step, 3200 molecules are generated, and molecules with predicted activity exceeding the probability threshold are admitted into the replay buffer. The replay buffer, in turn, influences training at the policy replay and fine-tuning steps. At the end of the training, the model generates 16,000 molecules for evaluation. We modified the number of iterations for all 3 steps in each epoch to understand their effects on training. We also used different libraries to initialize the replay buffer to understand how the replay buffer can influence model behavior.

Neural network optimization is a nontrivial task as network’s hyperparameter values define a training protocol. Owing to the high number of hyperparameters, the training space is vast. To complicate things further, neural network training is a computationally expensive task that can last hours to days. The choice of training hyperparameters thus has a significant influence on model quality. We sought to run a benchmark experiment to investigate how different training techniques interact and how they affect model quality. As a case study, we performed the optimization of the generative model with reinforcement learning to maximize the predicted probability of active class for EGFR protein. The experimental training pipeline is shown in Fig. 1.

Model training consists of two stages—pre-training the generator from scratch on a vast dataset such as ChEMBL^24^ in a supervised manner to produce mostly valid SMILES strings without any property optimization at this point. The second stage is training the model with RL to optimize the property values of the generated molecules. We used the pre-trained ChEMBL model and populated the experience replay buffer with generated predicted active molecules to initialize training. The model was trained using different combinations of policy gradient, experience replay, and fine-tuning. At the end of each substep, 3200 molecules were generated for intermediate evaluation. If experience replay and/or fine-tuning were used, molecules with predicted active class probability exceeding the probability threshold were admitted into the experience replay buffer. In turn, the replay buffer influences training at the policy replay and fine-tuning steps in the next epoch if used. At the end of the training, the model generated 16,000 molecules for evaluation. We first trained the model for a variable number of epochs and verified that the model learns significantly after 20 epochs (Fig. S2).

We used Random Forest ensemble model as a predictor in this pipeline. The ensemble model consists of five individual Random Forest models trained in a 5-fold cross-validation manner, and the final prediction is the mean of predictions from each model in the ensemble.

### Effect of fine-tuning vs. reinforcement learning

**Fig. 2:**
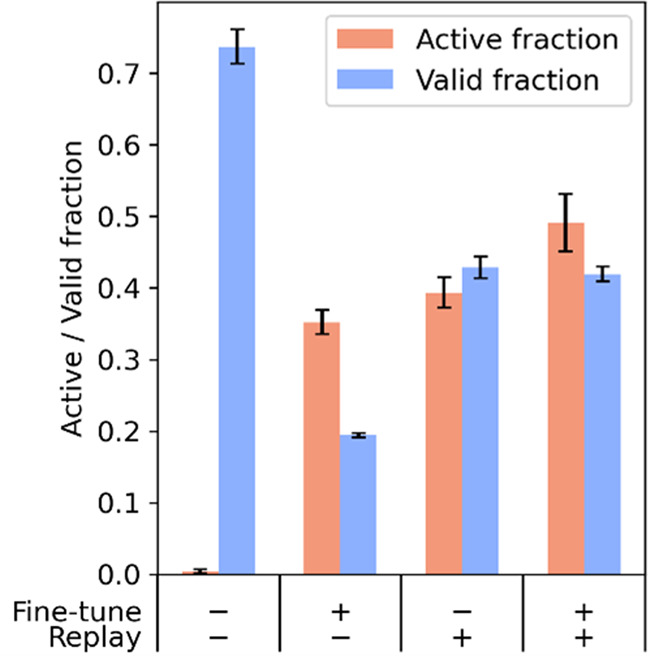
Combined effects of fine-tuning and reinforcement learning. Four conditions are shown here, representing the four combinations of fine-tuning and experience replay. From left to right, the conditions are: (1) no experience replay and no fine-tuning, (2) fine-tuning only, (3) experience replay only, and (4) both experience replay and fine-tuning. Models were trained for 20 epochs. Conditions with fine-tuning used 20 iterations of fine-tuning; those without used 0 iterations of fine-tuning. All training epochs had 25 policy steps, with different ratios of experience replay and policy gradient. Conditions with experience replay used 10 iterations of experience replay and 15 iterations of policy gradient; those without used 0 iterations of experience replay and 25 iterations of policy gradient.

The bar chart shown in Fig. 2 summarizes the findings for four representative conditions: (1) policy gradient only, (2) policy gradient and fine-tuning, (3) policy gradient and experience replay, and (4) policy gradient, experience replay, and fine-tuning. We assessed the extent of overfitting by recording the fraction of the generated trajectories that generate valid SMILES strings, which is defined as the ratio of valid and unique SMILES strings over the total number of the generated trajectories. In more detail, the model can overfit with respect to the property predictor. For example, if the QSAR model assigns high active class probabilities to molecules with a specific chemical group, the generative model can discover and exploit it by stacking multiple aforementioned chemical groups into a single molecule. Such scenario often leads to decrease in validity. We use ratio of valid and unique SMILES strings over the total number of generated trajectories. This metric would detect mode collapse, since we are discarding repeated molecules. We assessed the extent of model learning by recording the fraction of the generated trajectories resulting in active chemical structures, which is defined as the ratio of valid SMILES strings with predicted EGFR activity (with the arbitrary probability threshold of 0.75) over the number of valid and unique SMILES strings generated. Training without replay tricks has a near-zero “active” fraction and the highest “valid” fraction. This observation is consistent with the sparse rewards hypothesis. In the absence of rewards from active molecules, this model effectively trains on the classifier objective. Instead of learning to generate active molecules, the model optimizes valid fraction. Training with a single trick (fine-tuning or experience replay) teaches the model to generate active molecules, albeit at the expense of a lower valid fraction. Training with only fine-tuning results in a lower fraction of valid molecules. Training with both experience replay and fine-tuning yields the best results, with both high active fraction and high valid fraction. A more detailed summary with nine different training conditions is shown in Figs. S3 and S4. Figure S8 shows evolution of active and valid fractions over training.

Next, we analyzed the effect of fine-tuning steps on mode collapse^31^. Mode collapse poses a significant challenge in generative models. Reinforcement learning teaches generative models to produce output with high reward; however, it does not consider the distribution of generated output. Thus, the model can discover a pathological local minimum in the objective function by converging to generate a few instances with high reward; in such cases, the model undergoes mode collapse. Such overfitted models explore limited regions of chemical space and are undesirable for library generation.

Our experiments used the active fraction as a proxy for training progress and the valid fraction as a proxy for mode collapse. Two scenarios can decrease valid fraction: (1) the model generates a larger fraction of invalid SMILES strings (fewer valid SMILES strings), or (2) the model suffers from mode collapse and generates many repeats of the same SMILES string (fewer unique SMILES strings). The first factor is caused by the restricted chemical space of higher activity molecules and is specific to the reward function. The second factor is caused by the nature of training and can be controlled.

### Mode collapse effect

**Fig. 3:**
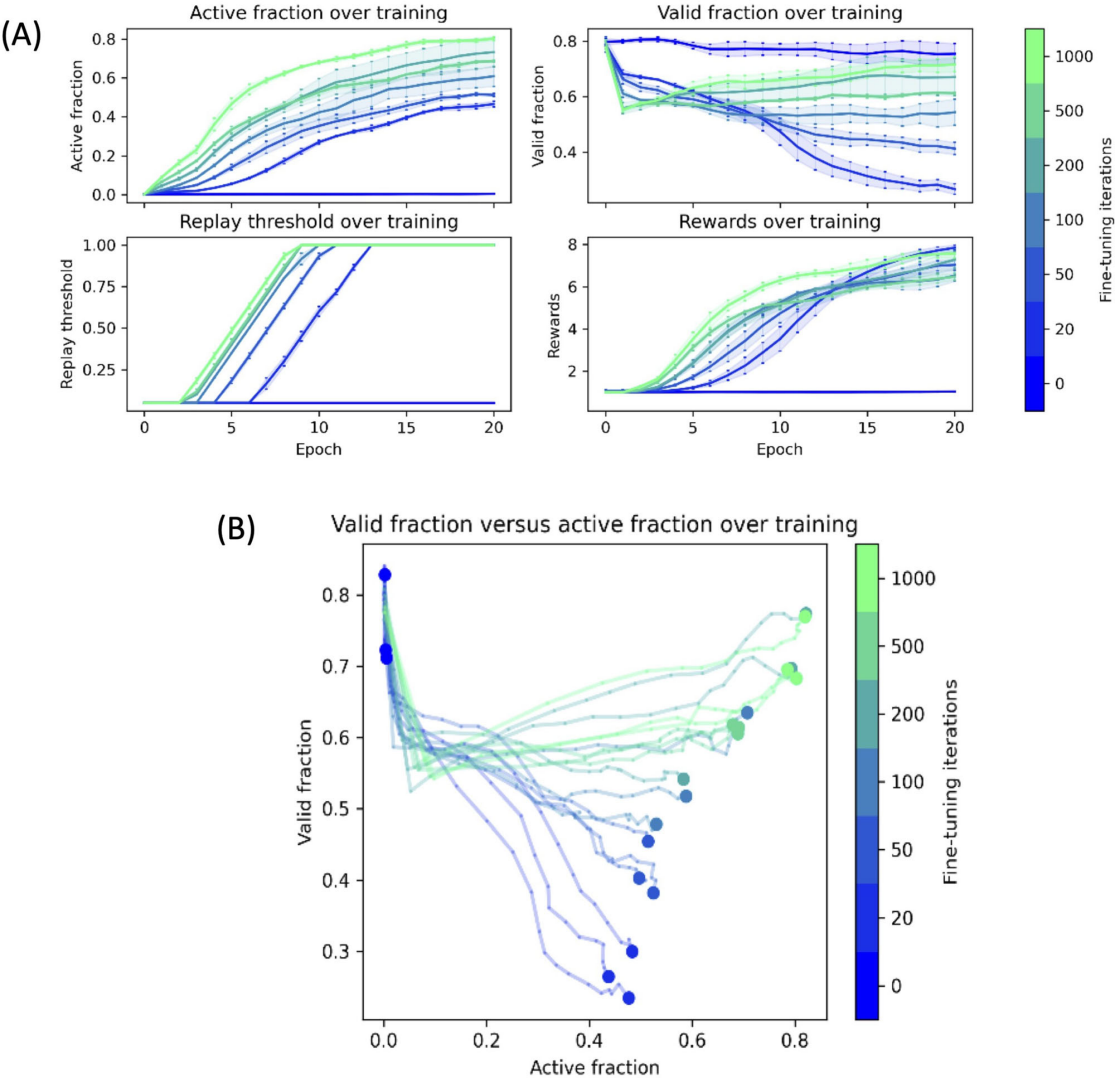
Training trajectories. **A** Trajectory of training for different fine-tuning values. Models were trained with 25 iterations of policy gradient and 0, 20, 50, 100, 200, 500, or 1000 iterations of fine-tuning per epoch. **B** Evolution of active and valid fractions over training. Models were trained with 25 iterations of policy gradient and 0, 20, 50, 100, 200, 500, or 1,000 iterations of fine-tuning per epoch. Solid lines represent training, small dots represent data at each epoch, and large dots represent data from the fully trained model. Graphs are color-coded by the number of fine-tuning iterations used per epoch.

To investigate how learning affects mode collapse, we ran several experiments where the generative model was trained with 25 iterations of policy gradient and one of 0, 20, 50, 100, 200, 500, or 1000 iterations of fine-tuning per epoch. We recorded valid fraction and active fraction after each epoch. The resulting trajectories are illustrated in Fig. 3. Figure 3A shows how active fraction, valid fraction, replay threshold, and average reward change with training for a different number of fine-tuning steps used in training. Figure 3B shows the joint trajectories of an active fraction and valid fraction change with training for the different number of fine-tuning steps.

Figure 3 shows that when the model uses no fine-tuning, it fails to produce active molecules and maintains a high valid fraction. When the model uses fine-tuning, it learns to generate active molecules at the expense of a lower valid fraction. All runs with fine-tuning experienced a significant drop in a valid fraction in the first epoch of training. This drop may represent a transient phase when the model cannot generate active molecules and partially overfits to the initial molecules in the replay buffer. The decrease in the valid fraction is more pronounced in models that use more fine-tuning iterations, consistent with this proposal. Models with the fewest fine-tuning iterations have the lowest active fraction and the lowest valid fraction. Over model training, the active fraction is negatively correlated with the valid fraction, suggesting that the model suffers mode collapse as it learns to generate active molecules. Models with higher fine-tuning iterations have progressively higher active fractions and valid fractions. The model appears to increase valid fraction for the highest numbers tested (500 and 1000 iterations) as it learns. Although models with higher fine-tuning iterations initially experience a more considerable drop in valid fractions, they eventually have higher valid fractions than models with lower fine-tuning iterations.

Similarly, we analyzed the effect of the different number of experience replay steps. All data is shown in Figs. S4 and S5. Similar to the fine-tuning benchmark, the model with no experience replay fails to generate active molecules and maintains a high valid fraction. Inclusion of experience replay results in successful learning with a simultaneous decrease in valid fraction. Unlike the fine-tuning benchmark, however, the number of experience replay steps does not clearly affect model quality. In these experiments, model quality is largely determined by the presence or absence of experience replay steps.

### Experience replay buffer effect

Finally, we investigated different initializations of the experience replay buffer. The experience replay library is typically filled with predicted molecules generated by the model pre-trained on the ChEMBL database, but our procedure enables us to use an arbitrary replay library alternatively. Owing to sparse rewards, model learning is initially dictated by the replay library. We generated a second replay library with molecules from the Enamine kinase library, which consists of 65,000 small molecules with predicted activity against kinases^32^. This library was chosen based on the expectation that general-purpose kinase inhibitors should contain scaffolds suitable for EGFR kinases.

We first selected molecules with non-zero active class probabilities for EGFR, as predicted by the random forest ensemble. We then filtered the active molecules to remove molecules with Bemis-Murcko scaffolds^33^ present in the historical EGFR data. This step ensured that the replay buffer molecules were dissimilar from known molecules. The final Enamine replay library had 219 molecules (Fig. S6).

**Fig. 4:**
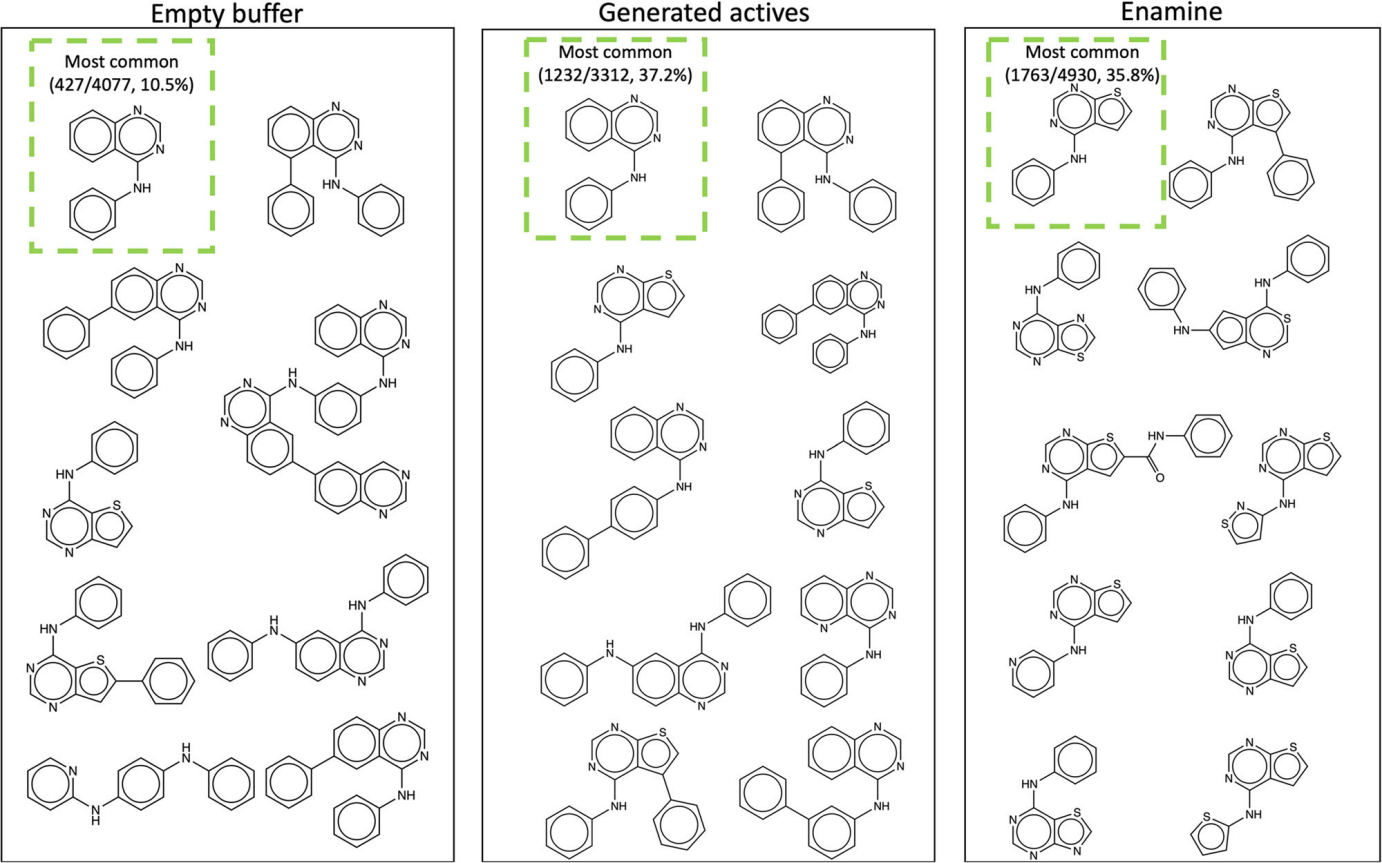
The 12 most common Bemis-Murcko scaffolds for models trained from different libraries. Three replay libraries were tested: an empty replay library (Empty buffer), the replay library from the model (generated actives), and the Enamine library selected as above (Enamine). Models were trained for 20 epochs with 15 iterations of policy gradient, 10 iterations of experience replay, and 20 iterations of fine-tuning per epoch. Scaffolds are sorted with decreasing counts from left to the right, then from top to bottom. The most common scaffolds had counts and percentages as follows: 427 out of 4077 predicted active molecules (10.5%) for the empty buffer, 1232 out of 3312 (37.2%) for the generated actives, and 1763 out of 4930 (35.8%) for the Enamine library.

This experiment tested three different replay libraries: an empty replay library (Empty buffer), the replay library from the model (Generated actives), and the Enamine library selected as above (Enamine). Figure 4 shows the 12 most common Bemis-Murcko scaffolds^33^ in the generated libraries produced by each of the models. All scaffold calculations were done using the RDKit^34^ package. Figure S5 also shows the 12 most common Bemis-Murcko scaffolds for replay libraries used in training.

In the generated library produced with replay buffer initialized with compounds from the Enamine kinase library, the main quinazoline scaffold is notably absent. The Enamine-trained library suffers from lower diversity, likely because the initial replay buffer selected from the Enamine kinase library predominantly contains thiophene-fused rings. Such bias was introduced by the predictive model used to select the initial replay buffer, as described in the Methods section. The predictive model favored compounds with thiophene-fused rings. This observation confirms that the initial selection of molecules in the replay library greatly influences the regions of chemical space that the model explores.

The library generated by the Empty buffer-trained model shows clear signs of overfitting, as 3 of the 12 most common scaffolds appear to be duplications of the quinazoline scaffold. The first active molecules greatly influence the model admitted into the replay library. When the replay library is initially empty, the model heavily exploits the first active molecules generated. As a result, the empty buffer-trained model explores a very limited region in chemical space (see Fig. S7 for similarity distributions).

### Generation and selection of hit compounds

**Fig. 5:**
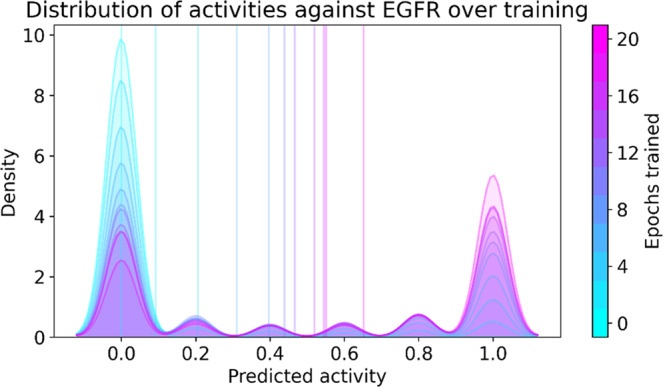
Time-lapse distribution of active class probability values during training. Mean predictions are marked by vertical lines. The model was trained for 20 epochs with 15 steps of policy gradient, 10 steps of experience replay, and 20 steps of fine-tuning per epoch. 1000 batches of molecules were generated every 2 epochs, and the distribution of active class probability plotted. Note that the classifier with ensemble size five generates discrete predictions of fifth fractions.

With the information obtained through computational analysis, we fixed the model training protocol. We trained the ChEMBL-pre-trained model for 20 epochs, with 15 steps of policy gradient, 10 steps of experience replay, and 20 steps of fine-tuning by transfer learning per epoch. Every 2 epochs, we produced snapshot libraries of 16,000 molecules. Each snapshot library included the distribution of active class probability for the generated molecules. Figure 5 illustrates the time-lapse of this distribution. The prominent peaks at 0 and 1 suggest that the model learns by increasing the fraction of highly active molecules, as opposed to generating molecules with progressively higher activities. This observation is likely because the random forest classifiers in the ensemble predictor were trained on the same dataset. Figure S7 also shows time-lapse distribution of Tanimoto similarities for libraries generated after different points in training.

### Experimental validation

With few notable exceptions^20,35^, most of the current de novo design publications are purely computational. However, it is important to know how many computationally predicted candidates are experimentally validated by in vitro (at least) assays. For this test, we established the following screening protocol.

The model described in the previous section was used to generate a large library of novel computational hits with high active class probabilities. To enable rapid testing of the computational models all hit molecules were parsed through the Enamine REAL database (Release 2020q1-2, https://enamine.net/library-synthesis/real-compounds/real-database) of 1.36B on-demand commercially available molecules. The Enamine REAL (readily accessible) database is based on the synthesis of ultra-large chemical libraries using two- or three-step three-component reaction sequences and available starting materials with pre-validated (at least 80% synthesis success rate) chemical reactivity^36^.

Seventeen computational hit molecules were matched with Enamine REAL. All of the predicted active compounds were derivatives of 4-anilinoquinazoline, a chemotype that was well represented in Enamine REAL (Table S1). The predicted active compounds contained a few small substituents on the quinazoline ring (positions 5–8: F, Cl, Br, OCH_3_) but a wide range of substituents on the 4-anilino group. As a negative control, we selected five molecules predicted to be inactive but containing the same 4-anilinoquinazoline scaffold (Table S1). The twenty three 4-anilinoquinazoline analogs were dissolved in DMSO and sent to Reaction Biology (https://www.reactionbiology.com/) for EGFR enzymatic assay screening. Two compounds in the predicted active series were insoluble in DMSO; therefore, biological tests were not performed. The 4-anilinoquinazoline analogs were initially tested in single-dose duplicate mode at a concentration of 1 μM and percent inhibition relative to DMSO control was determined (Table S1). Staurosporine was used as a reference EGFR tyrosine kinase inhibitor^37,38^.

Four 4-anilinoquinazolines from the predicted hit set showed >40% inhibition of EGFR enzyme activity in the 1 μM single-dose assay (Table S1), while all five of the negative control analogs were inactive. Notably, the four active compounds contained only small substituents (Br, NH_2_, CH_3_) at the 4’ position of the 4-anilino group (Table S1) paired with halogen substitution on the 5, 6, or 8 positions of quinazoline core. Surprisingly, however, the 4’-fluroanilino-6-fluroquinazoline analog was not active. Notably, all of the analogs with large linear or branched substituents at the 4’ position were inactive in the enzyme assay. The four active compounds from the single-dose assay were further tested in 10-dose IC_50_ mode with 3-fold serial dilution starting at 10 μM to determine their EGFR inhibition potency. The 4-anilinoquinazolines **1** and **2** (Table 1) were potent EGFR inhibitors with IC_50_ < 100 nM, comparable to the potency of staurosporine (Table S1). The 4-anilinoquinazolines **3** was slightly less potent with an IC_50_ = 210 nM. The analog **4** was the least potent with IC_50_ = 1.4 μM.

**Table 1:** Data for EGFR kinase inhibiton of compounds **1**–**4**.

Compound	Catalog ID	pIC50	Structure	Nearest neighbor (NN) from the training set	pChEMBL for NN
**1**	Z1192045732	7.5			7.6
**2**	Z1576525970	7.4			7.6
**3**	Z1182636554	6.7			7.3
**4**	Z1823625743	5.9			7.3
	Most active from ChEMBL				~10

Each of the active compounds **1**–**4** had a 3’-halogen substituted 4-anilinoquinazoline as a close neighbor in the training set that was reported to have a similar EGFR inhibition potency (Table 1). The most potent EGFR inhibitor from ChEMBL was N-(3-bromophenyl)quinazoline-4,7-diamine (CHEMBL420624), which had activity at sub-nanomolar concentrations. Although all five out of the negative control compounds were inactive in the EGFR enzyme assay, it should be noted that they each contain large linear or branched substituents at the 4’-position of the aniline. Analogs with the same or similar substitution on the aniline that were selected to be active in the computational model were also shown to be inactive in the EGFR assay (Table S1).

## Conclusions

### Summary of the study

Herein, we proposed several new improvements to the heuristics used to optimize properties of molecules created by generative neural networks with reinforcement learning and sparse rewards. Sparse rewards are commonly observed when maximizing the bioactivity of generated molecules for a specific target protein. Thus, classic reinforcement-learning algorithms such as policy gradient or Q-learning are not sufficient for such tasks. In contrast, our proposed tweaks, i.e., fine-tuning with transfer learning, experience replay, and real-time reward shaping, aim to extract informative feedback from the sparse reward signal and keep a healthy balance between exploration and exploitation. As a result of our study, we came up with a list of crucial points to consider when optimizing generative models with reinforcement learning.

1. We recommend considering the sparsity of the rewards and the desired level of balance between exploration and exploitation when selecting the right strategy for performing optimization in each case. The real-time reward shaping can be helpful in a sparse rewards scenario while unnecessary in cases when the reward feedback is sufficient (such as QED or LogP optimization).
2. The fine-tuning by transfer learning achieves a high level of exploitation, especially when used with known molecules. However, it will unlikely discover any chemotypes beyond the ones used for training.
3. The experience replay requires a rich and diverse pool of experience trajectories. Otherwise, this technique may also result in over-exploitation of replay examples. However, it can be a powerful tool to explore the chemical space and deal with sparse rewards in tandem with a policy gradient.

The optimized protocol was subject to a blind experimental validation. Out of fifteen tested compounds that were predicted active, four were confirmed in an EGFR enzyme assay. Two out of four compounds had nanomolar EGFR inhibition activity comparable to that of staurosporine. The overall hit rate was ~27%. Additionally, five compounds with the same scaffold as in active compounds but predicted as inactive were used as a negative control. All five compounds were confirmed as inactive. The obtained hit rate is *on par* with traditional virtual screening projects where molecule selection is guided by an expert medicinal chemist. However, in this work, we show that a properly trained AI model can mimic medicinal chemists’ skills in the autonomous generation of new chemical entities (NCEs) and selection of molecules for experimental validation. This is a prime example of the transfer of the decision power from human experts to AI. Such capabilities could be an important step toward true self-driving laboratories^39^ and serve as an example of the synergy between machine and human intelligence.

In summary, we do not think there is a current universal recipe for optimizing the properties of generated molecules with reinforcement learning. Each task is unique and requires thorough reward function engineering and hyperparameter search. However, as we have demonstrated with the EGFR inhibitor design example, with the right choice of the training protocol, generative models can be a powerful technique for automated and inexpensive de novo molecular design that can be executed even with limited computational and financial resources.

## Methods

In this section, we describe enhancements of deep-learning and reinforcement-learning approaches used to generate virtual molecules with desired properties. Briefly, we employ the reinforcement-learning pipeline introduced in our prior work^7^ with several improvements to overcome the problem of sparse rewards. Below we will talk about each part of the pipeline, introduce our proposed tricks and heuristics in more detail, and discuss an EGFR case study.

### Generative model

For the generative model, we used a deep-recurrent neural network with an augmented memory stack described in our previous work^7^. This network is trained to produce novel molecules in the form of SMILES strings^5^. The network has two modes—training mode and inference mode. In the training mode, the model receives a SMILES string from the training set and tries to reconstruct it, starting from the given prefix. The model is essentially trained as a multiclass classifier, where classes are represented as symbols in the SMILES string alphabet. In the inference mode, instead of receiving prefix from the training set, the model iteratively takes its output as new inputs to generate the next symbol based on the previously generated ones. The generation stops when the network produces a unique stop token interpreted as a command to end generation. The model is implemented as a part of OpenChem^40^ https://github.com/Mariewelt/OpenChem—an open-source deep-learning toolkit for computational chemistry and drug design.

### Reinforcement learning

For the method for shifting the distribution of predicted active class probability for generated molecules, we used the policy gradient algorithm^41^. We adapted the problem to a reinforcement-learning setting by treating the generative model as the policy network. In this formulation, the generative model predicts the probability of the next action, i.e., adding a new character to the SMILES string prefix. The set of actions is then limited to the SMILES alphabet. The set of states is then limited to all strings in the SMILES alphabet with lengths up to a specific limit *N*, where *N* is a hyperparameter defined by the maximum length of SMILES strings from the training dataset. According to the policy gradient algorithm, the objective function to be maximized is defined as the expected reward:

$${{{{{\rm{L}}}}}}\left({{{{{\rm{\theta }}}}}}\right)=-\mathop{\sum }\limits_{i=1}^{N}r ({s}_{N})\cdot {{{{{{\rm{\gamma }}}}}}}^{i}\cdot \log p\left({s}_{i} | {s}_{i-1}{{{{{\rm{;}}}}}}{{{{{\rm{\theta }}}}}}\right),$$

where ${s}_{N}$ is the generated SMILES string, ${s}_{i},{i}=1,...,{N}$ is the prefix of ${s}_{N}$ of length $0 \, < \, i \, < \, N,$ $\gamma$ is the discount factor, $p({s}_{i}|{s}_{i-1};\theta )$ is the transition probability obtained from the generative model, and $r({s}_{N})$ is the value of the reward function for the generated SMILES string based on the output of the predictive model of active class probability for EGFR.

### Exploration and exploitation trade-off

An encounter of a molecule active against a specific target (e.g., EGFR) is a rare event, so the generative model may very infrequently observe promising molecules. Such a scenario will result in over-exploration—a situation when the model mostly experiences low rewards for inactive molecules and receives insufficient signal to shift the distribution of the generated samples. At the same time, the model should not over-exploit information about known active molecules from the historical data, so that it can generate novel active molecules. We address this problem by complementing the classic policy gradient algorithm with heuristics detailed below to balance exploitation and exploration while training the model to maximize the predicted active class probability for the generated molecules.

(i) Fine-tuning by transfer learning on high-reward examples. The first algorithmic advance we have explored was to fine-tune the model by transfer learning using generated molecules with high rewards as training samples. Fine-tuning means training the model by minimizing cross-entropy loss in the same manner as during the pretraining stage. A similar idea has already been introduced in the literature^35^. Our approach differs from previous approaches through our selection process for fine-tuning training samples. Whereas the previous work uses historical data with high experimental activities, we used generated molecules as training samples. Overall, fine-tuning by transfer-learning results in high exploitation and low exploration. With sufficient rounds of fine-tuning, the generative model produces molecules highly similar to those used for fine-tuning. Thus, training on historical data results in the exploitation of already known chemical scaffolds instead of discovering novel scaffolds. Such an approach could be suitable for the lead optimization process when the goal is to optimize molecules with a prespecified scaffold. In contrast, fine-tuning on generated molecules with high rewards results in the exploitation of scaffolds produced by the generative network and highly scored by the predictive model. Generated scaffolds could be novel, thus increasing their potential in drug discovery applications.

(ii) Experience replay on high-reward molecules. Another technique that we proposed addresses the problem of sparse rewards while maintaining balancing the exploration-exploitation trade-off. To perform experience replay, we save high-reward trajectories (molecules) to the replay buffer. We randomly draw experience samples from the replay buffer during training and let the generative network follow the experience trajectory through teacher forcing^42^. We then calculate the expected reward maximization loss function and apply policy gradient updates to the generative network parameters. The concept of using experience replay for reinforcement learning is not new and has previously proven to be an effective training method in the reinforcement-learning domain^43–45^. We propose using this approach to deal with rare high-reward molecules while avoiding over-exploitation. Like the fine-tuning scenario, we utilize generated molecules with high rewards as training examples (or experiences) in the experience replay. Unlike the fine-tuning scenario, experience replay does not directly enforce specific characters in the generated SMILES string. Instead, it provides feedback in the form of a high reward at the end of the replay episode, resulting in less exploitation.

(iii) Real-time reward shaping. Real-time reward shaping is one more of our proposed advancements to train the neural network more efficiently in a situation when molecules with high rewards are observed rarely. The idea behind this technique is to change the reward function over training dynamically. We shall explain this concept using a threshold reward function and a predictive model returning the active class probability as an illustrative example. A molecule is considered active in these settings if the returned probability exceeds some threshold, such as 0.5. At the beginning of the training process, very few generated molecules will have such a high probability; instead, there often is a cohort of molecules with probabilities slightly higher than zero. The real-time reward shaping technique helps the model exploit molecules with non-zero predicted active class probabilities in the absence of good examples. We introduce the probability threshold ${p}_{0}$ to differentiate between good and bad examples in our threshold reward function:

$$R\left(s\right)=\left\{\begin{array}{c}{r}_{{{{{{{\mathrm{pos}}}}}}}},\,{{{\rm{if}}}}\,p\left(s\right) \, > \, {p}_{0},\\ {r}_{{{{{{{\mathrm{neg}}}}}}}},\,{{{{{{\mathrm{otherwise}}}}}}},\end{array}\right.$$

where $s$ is the generated molecule, $p(s)$ is the probability of active class returned by the predictive model, ${p}_{0}$ is the probability threshold, ${r}_{{{{{{{\mathrm{pos}}}}}}}}$ is the reward value for good examples, and ${r}_{{{\rm{{neg}}}}}$ is the reward value for bad examples. The probability threshold ${p}_{0}$ is initialized to a small value and dynamically increased during training. After several iterations of training, we generate a large enough batch of molecules with the current model and predict active class probabilities with the predictive model. The threshold ${p}_{0}$ is increased if the big enough portion of molecules has predicted active class probabilities bigger than the threshold’s current value. In our experiments, we started with ${p}_{0}=0.05$ and increased it by $0.05$ when at least $15 \%$ out of $3000$ generated molecules have predicted probabilities of active class greater than ${p}_{0}$.

### Case study

The generative model was pre-trained using the ChEMBL dataset^24^, which consists of ~2 million bioactive molecules. Notably, every molecule from ChEMBL has reported experimental bioactivity for at least one protein target. The pretraining step teaches the generative model to fit the distribution of molecules from the training data. Once pre-trained, the generative network is used to sample new molecules from this distribution. Thus, we can assume that pretraining on a dataset of bioactive molecules such as ChEMBL ensures that the generative model will be capable of sampling bioactive-like molecules. This feature is essential to us since our ultimate goal is to produce active molecules to inhibit EGFR.

### Activity data and predictive model

The predictive model was trained on historical experimental data of activities for EGFR extracted from ChEMBL. The EGFR training dataset includes bioactivities extracted from ChEMBL 25 (Target ID CHEMBL203). We considered only pChEMBL activities with a confidence score of 8 or greater for “binding” or “functional” human EGFR assays. Replicate compounds with bioactivity differences larger than one unit on a log scale were excluded. For similar replicate measurements, a single representative assay value was selected for inclusion in the training dataset. Activity values were binarized according to the 1 μM cutoff. Chemical data were processed using OpenEye chemistry toolkit^46^. Standardizer was used for structure canonicalization, JChem 18.2, 2018, ChemAxon (http://www.chemaxon.com). The dataset was curated according to a well-known protocol^47^.

For the predictive model, we used an ensemble of five random forest (RF) classifiers. For features, we used 2048-bit ECFP fingerprints as implemented in RDKit (https://www.rdkit.org/). We trained five random forest models on a cross-validated dataset to solve a binary classification problem. Each model in the ensemble returns the probability of class “active” for an input molecule. The resulting ensemble prediction is obtained by averaging predictions of all models in the ensemble.

An interesting observation about this dataset is the presence of a privileged scaffold. Around 50% of molecules that fall into active class after binarization contain quinazoline chemotype^48,49^, a known hinge binder in kinase inhibitors^50^. From the crystal structures of know EGFR inhibitors, it is known that hydrophobic residues surround quinazoline ring. The aniline group substituted at the 4th position of quinazoline ring and itself quinazoline ring of drugs like gefitinib and erlotinib are bounded by the hydrophobic pocket^51,52^. With such a 4-anilinoquinazoline prevalence, we expect to see a bias in the predictive model’s predictions towards this specific chemotype.

### Experimental validation

Compounds that emerged as computational hits were purchased from Enamine (https://www.enaminestore.com/) and resuspended in 100% DMSO at 10 mM concentration. In vitro experiments were performed at Reaction Biology (https://www.reactionbiology.com/) using a radioactive assay based on the transfer of ^33^P-labeled phosphate from ATP to the kinase substrate^53^. The HotSpot^SM^ assay utilizes a miniaturized filter binding, where reaction mixtures are spotted onto filter papers. Then reaction mixture binds the radioisotope-labeled catalytic product. Unreacted phosphate is removed via washing of the filter papers. All reactions were carried out at 10 μM ATP concentrations.

## Supplementary information

Korshunova_PR File

Supplementary material

## Data Availability

All data used in this study are publicly. Training data for the generative and predictive models were downloaded from ChEMBL (https://www.ebi.ac.uk/chembl/) as described in ref. ^7^. (EGFR Target ID CHEMBL203). Enamine kinase library was downloaded from Enamine website (https://enamine.net/compound-libraries/targeted-libraries/kinase-library).

## Code availability

The code to reproduce this study is available on GitHub at https://github.com/isayevlab/rl_experiments
