# Supplementary material for: Generative and reinforcement learning approaches for the automated de novo design of bioactive compounds
Source: Commun Chem. 2022 Oct 18;5:129. doi: 10.1038/s42004-022-00733-0 (PMC9814657; doi:10.1038/s42004-022-00733-0)
Supplement: Supplementary file 2 — Supplementary material [file 42004_2022_733_MOESM2_ESM.pdf]

Generative and reinforcement learning approaches for the automated de novo design of bioactive compounds

Maria Korshunova[1,2], Niles Huang[3], Stephen Capuzzi[4], Dmytro S. Radchenko[5,6], Olena Savych[5], Yuriy S. Moroz[6,7], Carrow I. Wells,[8] Timothy M. Willson[8], Alexander Tropsha[4], Olexandr Isayev[1,2*]*

[1] Department of Chemistry, Mellon College of Science, Carnegie Mellon University, Pittsburgh, Pennsylvania, United States of America
[2] Computational Biology Department, School of Computer Science, Carnegie Mellon University, Pittsburgh, Pennsylvania, United States of America
[3] Department of Biochemistry, University of Oxford, Oxford, United Kingdom
[4] Laboratory for Molecular Modeling, UNC Eshelman School of Pharmacy, University of North Carolina at Chapel Hill, Chapel Hill, North Carolina, United States of America
[5] Enamine Ltd, 78 Chervonotkatska Street, 02094, Ukraine
[6] Taras Shevchenko National University of Kyiv, Volodymyrska Street 60, Kyiv 01601, Ukraine
[7] Chemspace LLC, Chervonotkatska Street 85, Suite 1, Kyiv 02094, Ukraine
[8] Structual Genomics Consortium, UNC Eshelman School of Pharmacy, University of North Carolina at Chapel Hill, Chapel Hill, North Carolina, United States of America
[*] Correspondence: mariewelt@cmu.edu (M.K.); olexandr@olexandrisayev.com (O.I.)

Supplementary material

Table S1. EGFR enzyme inhibition of 4-anilinoquinazolines

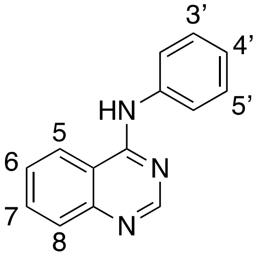

Predicted actives

Compound	5	6	7	8	3’	4’	5’	EGFR % I at 1 μM	EGFR pIC_{50}
ZINC000092611293	H	F	H	H	H	F	H	12	
ZINC000000099087	H	Br	H	H	H	F	H	96	7.5
ZINC000302791534	H	H	H	F	Cl	NH_2	Cl	47	5.9
ZINC000301931984	H	F	H	H	Cl	NH_2	Cl	89	7.4
ZINC000480349471	F	H	H	H	F	CH_3	H	83	6.7
ZINC000302555896	H	OCH_3	F	H	F	$OCH_2CH_2OCH_3$	H	12	
ZINC000480418233	F	H	H	H	F	$OCH_2CH_2OCH_3$	H	8	
ZINC000605398532	H	H	Cl	H	H	$OCH_2CH(OH)(CH_3)_2$	H	18	
ZINC000301175853	H	H	Cl	H	H	$OCH_2CH_2CONHCH_3$	H	12	
ZINC000301950472	H	F	H	H	H	$CH_2CH_2N(CH_3)_2$	H	10	
ZINC000301275665	H	H	Cl	H	H	$CH_2N(Ac)cPr$	H	—	
ZINC000041479925	H	F	H	H	H	$N(CH_2CH_2CH_2CH_2)$	H	8	
ZINC000089266948	H	F	H	H	H	$N(CH_2CH_2CH(N(CH_3)_2)CH_2CH_2)$	H	14	
ZINC000302845052	H	H	H	F	H	$N(CH_2CH_2CH(N(CH_3)_2)CH_2CH_2)$	H	12	
ZINC000301979927	H	Br	H	H	H	$N(CH_2CH_2CH(N(CH_3)_2)CH_2CH_2)$	H	8	
ZINC000261436963	H	Br	H	H	H	$N(CH_2CH_2CH(CONH_2)CH_2CH_2)$	H	—	
ZINC000737413605	H	Br	H	H	H	$N(CH_2CH_2CH(CH_2N(CH_3)_2)CH_2CH_2)$	H	14	
Staurosporine								100	7.1

Enzyme inhibition in the presence of 10 μM ATP. —, not tested due to poor solubility.

Predicted inactives

Compound	5	6	7	8	3’	4’	5’	EGFR % I at 1 μM	EGFR pIC50
ZINC000572311742	H	H	Cl	H	H	$OCH_2CH_2CH_2OH$	H	0	
ZINC000302873009	H	H	H	F	Cl	$OCH_2CH_2OCH_3$	H	16	
ZINC000440544230	H	F	H	H	H	$O(cC_6H_{10}\text{-}2\text{-}OH)$	H	4	
ZINC000594704454	H	Cl	H	H	H	$CH_2CH_2N(CH_3)_2$	H	8	
ZINC000112964683	H	Br	H	H	H	$N(CH_2CH_2CH(OH)CH_2CH_2)$	H	10	

Enzyme inhibition in the presence of 10 µM ATP.

QED score distributions

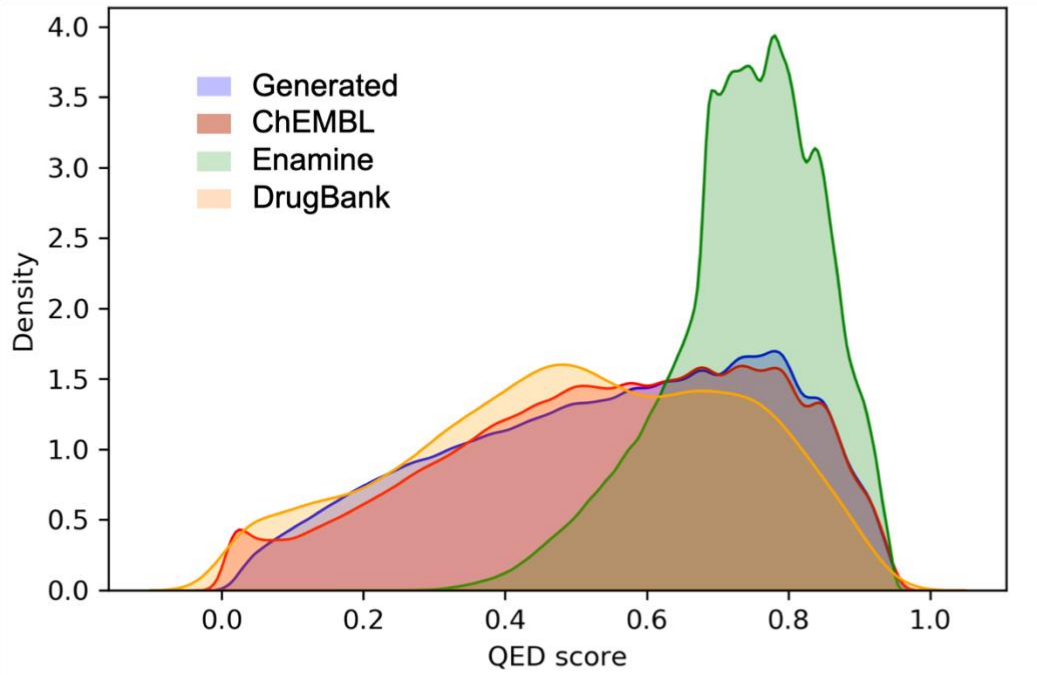

Figure S1. Distributions of QED score for molecules from various sources – generated with model pre-trained on ChEMBL, ChEMBL, Enamine REAL, DrugBank. Each distribution is computed based on 10000 randomly sampled molecules

Summary of replay trick results, extended

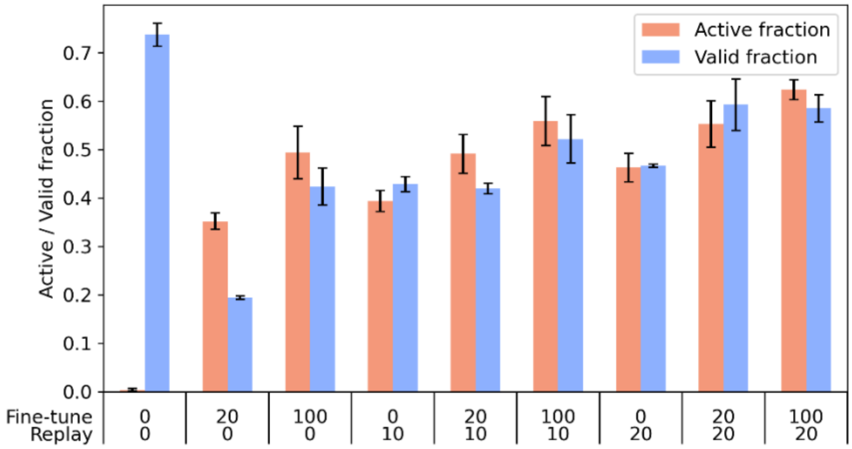

Figure S2. Combined effects of fine-tuning and reinforcement learning. Models were trained for 20 epochs for nine different combinations of fine-tuning and experience replay with the following options: no fine-

tuning, 20 iterations of fine-tuning, or 100 iterations of fine-tuning; and no experience replay, 10 iterations of experience replay, and 20 iterations of experience replay. The number of policy gradient steps was adjusted so that each training epoch had 25 iterations of replay and policy gradient (e.g. 25 policy steps for 0 replay steps and 15 policy steps for 10 replay steps).

Replay ratio benchmark

Models were trained with experience replay only. Each training condition used 25 iterations policy steps: either 25 and 0, 5 and 20, 10 and 15, 15 and 10, or 20 and 5 iterations of policy gradient and experience replay, respectively. Similar to the fine-tuning benchmark, the model with no experience replay (25 and 0 iterations of policy gradient and experience replay, respectively) fails to generate active molecules and maintains a high valid fraction. Inclusion of experience replay results in successful learning with simultaneous decrease in valid fraction. Unlike the fine-tuning benchmark however, the number of experience replay steps does not have a clear effect on model quality. In these experiments, model quality is largely determined by the presence or absence of experience replay steps.

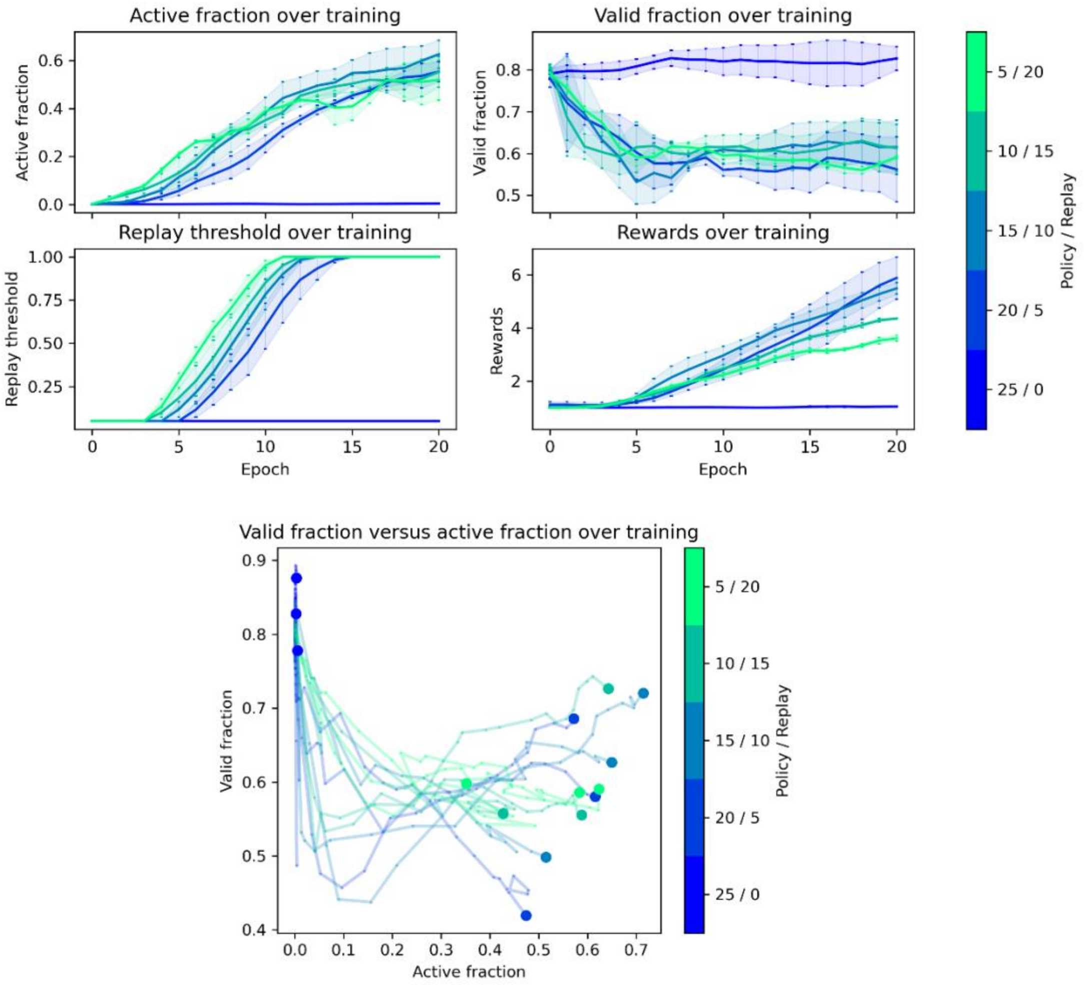

Figure S3. Evolution of active and valid fractions over training. Each training condition used 25 policy steps per epoch: either 25 and 0, 5 and 20, 10 and 15, 15 and 10, or 20 and 5 iterations of policy gradient and experience replay, respectively. Solid lines represent training, small dots represent data at each epoch, and large dots represent data from the fully-trained model. Graphs are color-coded by the number of experience replay steps used per epoch.

Varying replay ratio, with fine-tuning kept constant

To see how fine-tuning and experience replay interact, models were trained with both techniques. Each training condition used 20 iterations of fine-tuning and 25 iterations policy steps: either 25 and 0, 5 and 20, 10 and 15, 15 and 10, or 20 and 5 iterations of policy gradient and experience replay, respectively. Unlike the experiment with experience replay and no fine-tuning, this experiment shows a graded response to the number of experience replay steps. The condition with no experience replay steps performs worst, with the lowest active fraction and severe overfitting, as indicated by a low valid fraction. The condition with 5 replay steps and 20 policy gradient steps produces both higher valid and active fractions. The other three conditions perform even better, although the differences between them are small.

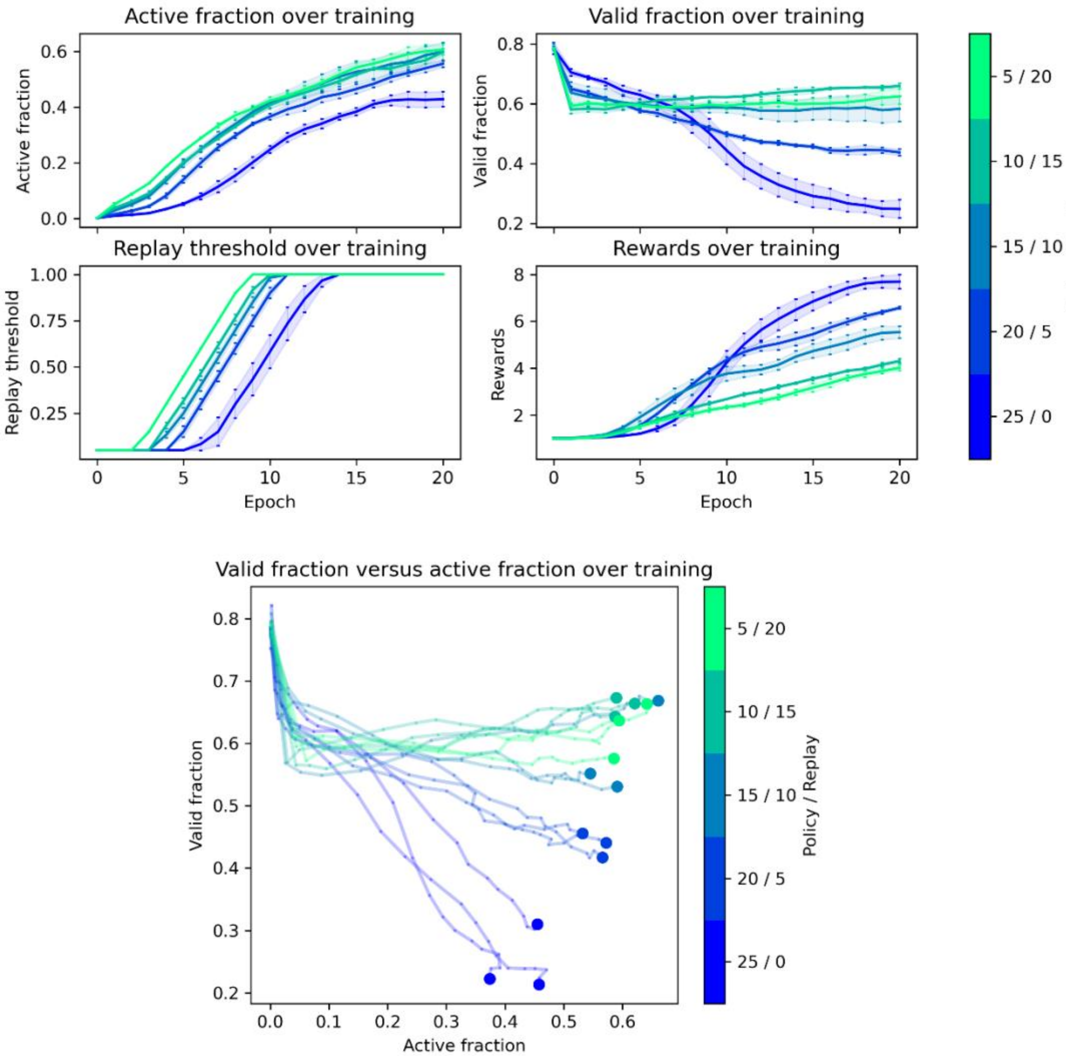

Figure S4. Evolution of active and valid fractions over training. Each training condition used 20 iterations of fine-tuning and 25 policy steps per epoch: either 25 and 0, 5 and 20, 10 and 15, 15 and 10, or 20 and 5 iterations of policy gradient and experience replay, respectively. Solid lines represent training, small dots represent data at each epoch, and large dots represent data from the fully-trained model. Graphs are color-coded by the number of experience replay steps used per epoch.

Replay library

The most common scaffolds in the replay libraries used for training are shown below. The generated actives library consists of molecules generated by the pre-trained model. 160,000 molecules were generated by the ChEMBL-trained molecule, and 216 molecules had non-zero predicted activities against EGFR. These molecules were admitted into the generated actives library. This library has a high proportion of quinazoline scaffolds, as well as a smaller proportion of thiophene-fused rings.

The Enamine replay library consists of molecules from the Enamine kinase library. We first selected molecules with nonzero activities against EGFR, as predicted by the random forest ensemble. We then filtered these molecules to remove Murcko scaffolds present in the experimental EGFR library. This step ensured that the replay buffer molecules were dissimilar

from molecules known to be active. The final Enamine replay library had 219 molecules. This library has a high enrichment of thiophene-fused rings. This is likely because the filtration step removes molecules with quinazoline scaffolds known to be active; nevertheless, even the non-filtered Enamine library has a high occurrence of thiophene-fused rings.

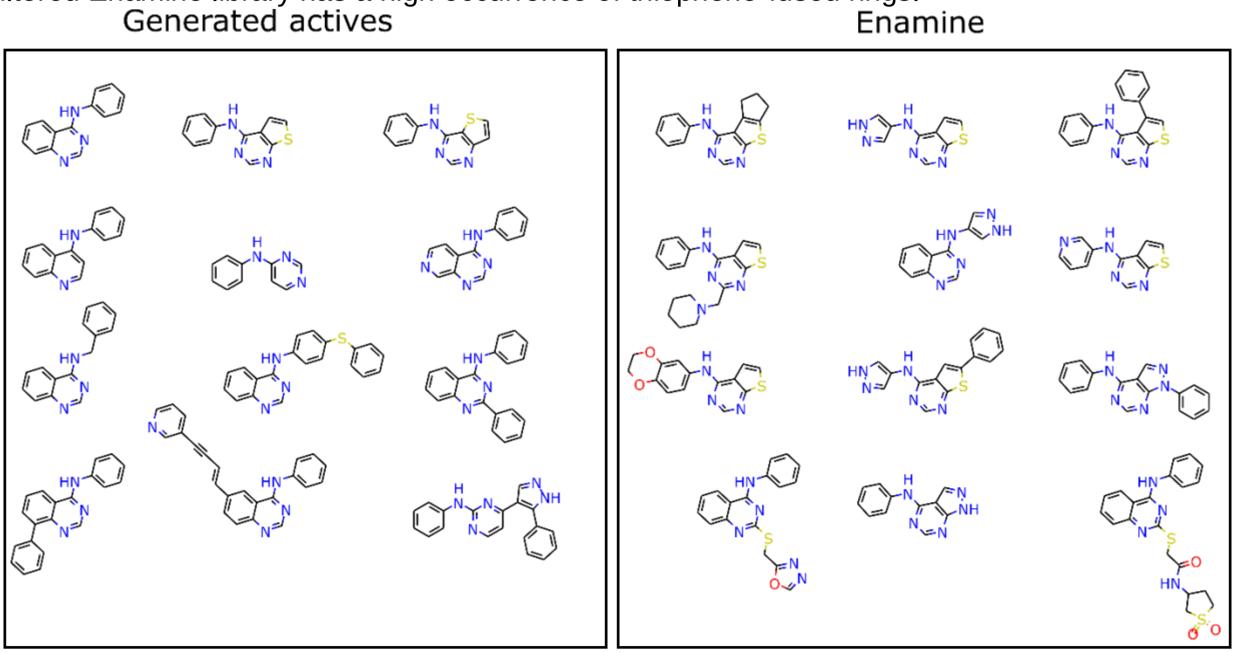

Figure S5. The 12 most common Murcko scaffolds for replay libraries used in training. Two replay libraries were used in training: a generated actives library, and an Enamine replay library. Scaffolds are sorted with decreasing counts from left to right, then from top to bottom. The most common scaffolds had counts and percentages as follows: 22 out of 216 predicted active molecules (10.2%) for the generated actives library, and 11 out of 219 (5.02%) for the Enamine replay library.

Replay buffer similarity distributions

Models were trained using one of three replay libraries: an empty replay library (empty buffer), the replay library from the model (generated actives), and the enamine library selected as above (Enamine). The distributions of Tanimoto fingerprint similarities for the active molecules (probability exceeding 0.75) of each library are shown below. The generated libraries for the empty buffer and the Enamine replay buffer have high similarities, which suggests some degree of overfitting.

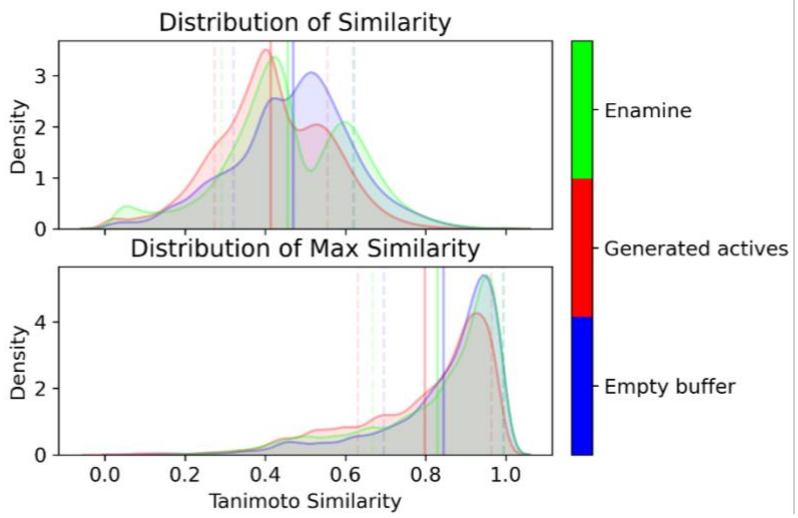

Figure S6. Distributions of Tanimoto similarities for libraries generated after training on different replay buffers. Distribution means are marked by vertical solid lines, and distribution standard deviations from the mean are marked by vertical dashed lines. The distribution of pairwise fingerprint similarities provide a global picture of library diversity. The distribution of maximum fingerprint similarities represents the distribution of nearest neighbors and informs on the local density around each molecule.

Timelapsed evolution of libraries

To investigate the progress of model training, we modified the training procedure to produce 'snapshot' libraries of 16,000 molecules every 2 epochs for 20 epochs of training. The distributions of similarities were then calculated for each library. Over training, the generated libraries have higher similarities. As the model learns to generate active molecules, it generates molecules from a restricted chemical space of active molecules.

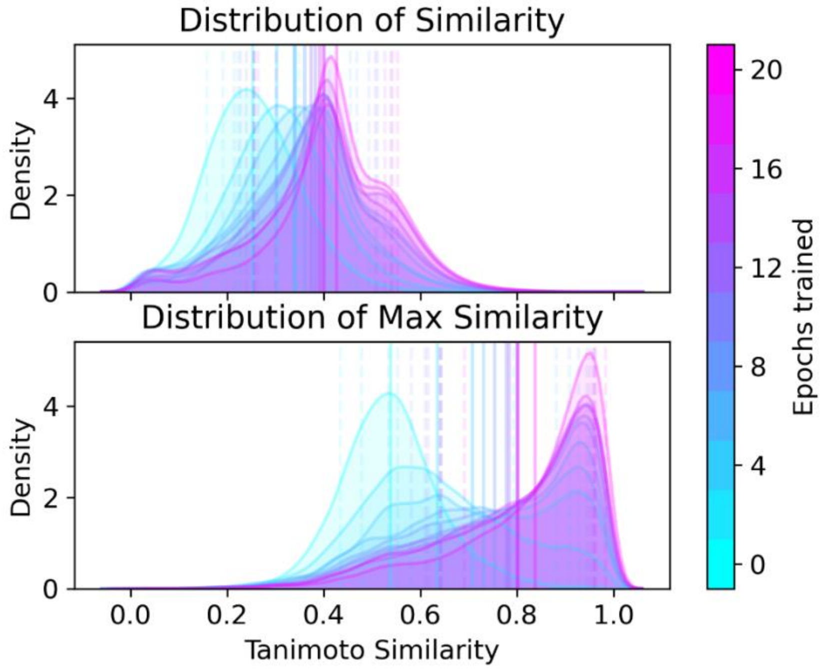

Figure S7. Distributions of Tanimoto similarities for libraries generated after different points in training. Models were trained for 20 epochs, with 15 iterations of policy gradient, 10 iterations of experience replay, and 20 iterations of fine-tuning per epoch. Distribution means are marked by vertical solid lines, and distribution standard deviations from the mean are marked by vertical dashed lines. The distribution of pairwise fingerprint similarities provide a global picture of library diversity. The distribution of maximum fingerprint similarities represents the distribution of nearest neighbors and informs on the local density around each molecule.

Number of iterations

Models were trained for different numbers of epochs to see how the model behaves in response to overtraining. The model was trained with 20 iterations of fine-tuning, 15 iterations of policy gradient, and 10 iterations of experience replay, for either 10, 20, 50, or 100 epochs. The model learns significantly by 20 epochs, and further training steps gradually show reduced margins for

active fraction. Interestingly, the model maintains high valid fraction and active fraction at high epochs, suggesting that the model is robust to training steps.

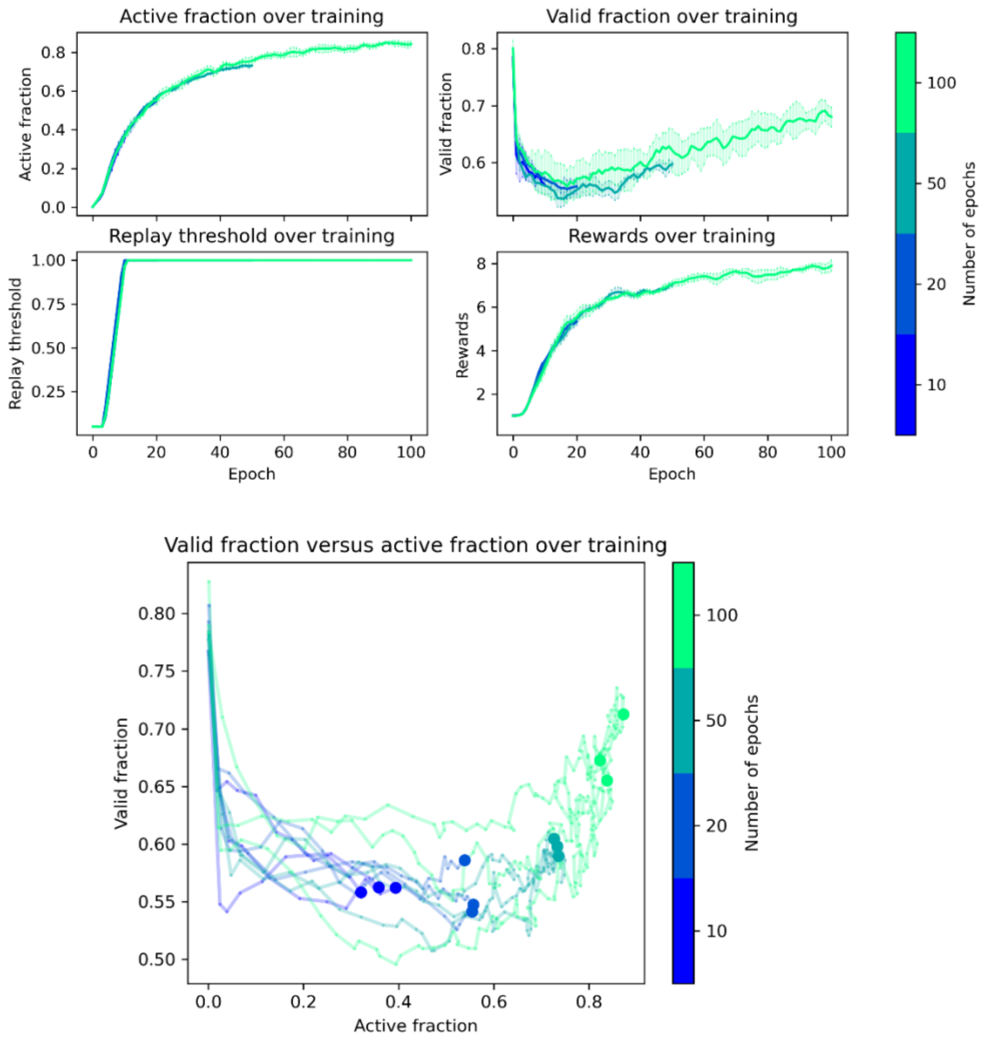

Figure S8. Evolution of active and valid fractions over training. Each training condition used 15 iterations of policy gradient, 10 iterations of experience replay, and 20 iterations of fine-tuning per epoch. Models were trained for 10, 20, 50, or 100 epochs. Solid lines represent training, small dots represent data at each epoch, and large dots represent data from the fully-trained model.
