# Supplementary material for: Generative and reinforcement learning approaches for the automated de novo design of bioactive compounds
Source: Commun Chem. 2022 Oct 18;5:129. doi: 10.1038/s42004-022-00733-0 (PMC9814657; doi:10.1038/s42004-022-00733-0)
Supplement: Supplementary file 1 — Korshunova_PR File [file 42004_2022_733_MOESM1_ESM.pdf]

nature portfolio

Peer Review File

Generative and reinforcement learning approaches for the automated de novo design of bioactive compounds

Open Access This file is licensed under a Creative Commons Attribution 4.0 International License, which permits use, sharing, adaptation, distribution and reproduction in any medium or format, as long as you give appropriate credit to the original author(s) and the source, provide a link to the Creative Commons license, and indicate if changes were made. In the cases where the authors are anonymous, such as is the case for the reports of anonymous peer reviewers, author attribution should be to 'Anonymous Referee' followed by a clear attribution to the source work. The images or other third party material in this file are included in the article's Creative Commons license, unless indicated otherwise in a credit line to the material. If material is not included in the article's Creative Commons license and your intended use is not permitted by statutory regulation or exceeds the permitted use, you will need to obtain permission directly from the copyright holder. To view a copy of this license, visit http://creativecommons.org/licenses/by/4.0/.

Reviewers' comments:

Reviewer #1 (Remarks to the Author):

This manuscript by Korshunova et al. reports heuristics for fine-tuning the performance of reinforcement-learning based molecular optimization approaches and the experimental validation of some computer-generated leads.

The work is interesting and the authors go well above and beyond by adding experimental validation to a very computational paper, which is rare and commendable. However, the manuscript suffers from this "split personality". The computational analysis is a relatively exhaustive exploration of model choices and hyperparameters. It is valuable and insightful but not intellectually surprising. To some degree, it is hard labor and clear performance metrics, but mostly journeyman in its vision.

Then the experimental validation really stands out among related papers, but is unfortunately not enough to carry the paper. The model predictions are fine but not groundbreaking (all kinase inhibitors look very much alike) and the models re-discovers chemistry that is not very surprising. Because custom synthesis is out of scope, the authors are forced to order from virtual catalogs, which they could have done by just screening the catalog with forward models.

Lastly, the language, the notation and the prose lack rigor some times.

In summary, i think this work may have all the building blocks for success, but they need to be better integrated and demonstrated.

Technical comments.
"Moreover, a naïve generative model pre-trained on a dataset of drug-like compounds such as ChEMBL23 would produce molecules with relatively high QED values" That may be the expectation - that a model retains the characteristics of the datasets it was trained on -, but is not necessarily the case and particularly not for synthetic accessibility in RL works. Is it possible to support this statement with literature references? Refs 10,20,24 report optimizing QED, not that high QED from the training data translated to the generated molecules.

"In contrast to physical properties such as LogP that can be calculated directly from molecular structure" LogP is not calculated from structure through some physics law. It is predicted through fitted QSAR or ML model, much in the same way as a binding affinity is. The authors perhaps mean that the latter model has less training data than the former or that it's more non-linear.

"However, unlike physical molecular properties like LogP that every molecule possesses, specific bioactivity is a target property that exists for only a small fraction of molecules, which leads to the reward sparseness in the generative models" This might be just poor phrasing, but all molecules will show binding affinity (most of them a very small one) towards any target. The statement is perhaps more applicable to classifiers, since mediocre and extremely bad binders all get the "negative" label. Still, a QSAR classifier will predict a continuous output. The discussion should disentangle sparseness _in the magnitude itself_ with sparsity in the availability of labeled data.

None of the statements in line 82 to 91 are supported with literature references.

"Training the generative network to optimize the potency of " Potency is a continuous variable that is in principle amenable to a continuous reward function. The previous comment stands: is this a data quantity problem? Or a problem of the nature of the measured variable?

"could not discover any active molecules for EGFR due to sparse reward" How were molecules

validated to be active?

" generated molecules with high active class probabilities" I encourage the authors to use consistent language to refer to the outcomes of the approach. Is this equivalent to "active molecules" above?

"As described above, we used the pre-trained ChEMBL model " Above where? The caption of Figure 1? "The model was pre-trained on ChEMBL data and then trained for 20 epochs." What is the difference between pre-train and train? It is not stated. Lines 149 - 155 repeat information in the caption.

"We assessed the extent of overfitting by recording the fraction of the generated trajectories that generate valid SMILES strings, which is defined as the ratio of valid and unique SMILES strings over the total number of the generated trajectories" Why is overfitting related to validity? Couldn't it also result in mode collapse?

"with the arbitrary probability threshold of 0.75" A lot seems to be hinging on this choice. How is it justified? Does it modify the results if it changes? Which fraction of the activity test data is under this threshold?

"the valid fraction as a proxy for mode collapse. Two scenarios can decrease valid fraction: " Why not disentangle those two? Make one metric for unique strings and another for their validity? The text below suggests this partitioning is applicable.

"as predicted by the random forest ensemble" What random forest ensemble? This hasn't been mentioned yet. The model should be introduced when it's first used. (line 165? or earlier)

"remove molecules with Bemis Murcko scaffolds present in the historical EGFR data" This may be insufficient for heterocycles, where some C =>N or S (or vice versa) substitutions in a ring may switch scaffolds but not chemical similarity. I encourage the authors to replicate this with Tanimonoto-similarity spherical clustering instead of / in combination with the scaffold splits.

Figure 4 Caption. " The most common scaffolds had counts and percentages as follows" Why not just write the count under the scaffold as a number - percentage tuple?

Figure 5. x axis say "predicted activity" In the same line as above, i encourage the authors to be more rigorous around what it is that the model is predicting. Is it a score, a probability, an activity ? Line 291-292 "progressively higher activities" The model does not predict activity, it predicts the likelihood of being active. There is no reason to assume higher likelihood implies higher potency.

" With few notable exceptions, most of the current de novo design publications are purely computational" I wholeheartedly agree, and this is a tremendously strong point in favor of this work.

Ln 306 "high active class probabilities" The notation keeps changing.

Ln 310 "synthesis of ultra-large chemical libraries" These libraries have not been synthesized. Enamine REAL is based on synthesizing arbitrary members of such virtual libraries.

Why are there only 4 molecules in Table 1 if 23 were ordered? It is important to show the full story. What other candidates were ordered but not active (maybe not the insoluble ones) ? How chemically dissimilar to the positives and to the training data were the negative controls? Are they truly a challenge or obvious inactive even to the eye. What about the 11 false positives? All this belongs in the main paper, IMO. What is meant by NN in the training set. Is this in the random forest training set or in the generative model training set?

"Notably, the four active compounds contained only small substituents (Br, NH2, CH3) at the 4' position of the 4-anilino group (Table S1) paired with halogen substitution on the 5, 6, or 8 positions of quinazoline core" Just like the NN in the training set?

"Surprisingly, however, the 4′-fluroanilino-6- fluroquinazoline analog was not active" All the more reason to bring these comparisons to the main text

"Notably, all of the analogs with large linear or branched substituents at the 4' position were inactive in the enzyme assay" What about their NNs? Were they labeled as positive or negatives?

How would the approach compare in terms of computational cost with running an exhaustive search over Enamine REAL with the random forest model?

Minor language comments
"application of the deep generative recurrent neural network " This seems to be missing a word. The deep generated RNN _architecture_ ?

"enhanced by several novel technical tricks to designing experimentally validated potent inhibitors of the epidermal growth factor (EGFR)." This sentence is ambiguous - one cannot design experimentally validated compounds, typically one designs the compounds and then validates. The authors should clarify whether this is a prospective or retrospective study.

"the success rate of finding novel bioactive compounds" Finding already implies it was a success

"molecule's SMILES"

"Problem of sparse rewards in reinforcement learning" missing the definite article.

"novel approach for discovering novel bioactive molecules"

"Neural network training is a nontrivial task as its hyperparameter values" hyperparameters of the task, the training or the network?

Lines 127-131 seem overly repetitive.

Reviewer #2 (Remarks to the Author):

The paper of M. Korshunova et al. reports a set of techniques - 1) fine-tuning, 2) experience replay, and 3) real-time reward shaping - applied in de novo design task named "a bag of tricks". As the authors mention, techniques 1 and 2 have been already proposed earlier, and their extension is demonstrated in the paper, whereas technique 3 is new. The authors conclude that the application of these techniques in combination with the reinforcement-learning approach improves the active molecules generation process for the given Neural Network architecture. The selected generated molecules were tested, and 2 compounds with an IC50 lower than 100 nM were discovered. This shows the importance of the study and the readiness of the proposed architecture to be used in a real drug discovery project. Still, some questions arise:
1) Is there a chance to do something similar for a graph-based model, and is there any sense to go there?
2) line 79 - it is better to replace "binary outputs" with "categorical outputs" to respect the multi-class classification tasks.
3) Figure 3 - Letters (A) and (B) are given in the caption but there is no such in the figure.
4) lines 418-420 - invisible symbols.

5) lines 436-437 - The fine-tuning procedure was modified by moving from experimental to predicted values. Shouldn't this introduce some noise or "model's quality" bias to the results of the transfer learning?
6) It is not clear whether compounds predicted as highly active are mixed with others in the experience replay or not?
7) line 249 - the meaning of the "non-zero actives" phrase is not clear. I assume it has the same meaning as "non-zero predicted probabilities" mentioned in lines 469-470, thus, it should be clarified.

Nevertheless, the paper is of good quality and it worths to be published.

Reviewer #3 (Remarks to the Author):

The authors present a study on the application of reinforcement learning and generative modeling to the use case of designing active inhibitors against EGFR, a key protein in various cancer types.
The work presents in a clear fashion a series of "tricks" and techniques that can be exploited to improve known issues of molecular design, especially the sparse reward dilemma, that usually hinders the ability to explore relevant regions of the molecular space, hence limiting the capacity to generate/design consistently active candidates.

I believe that the paper can be considered ready for publication even in the current form, but here I leave some comments that I think could help in expanding the discussion and increase the, already high, quality of the work.

Always within the realm of RL applications to molecular design, another recent work alleviated the sparsity of the reward by considering a multi-modal predictor that acts on fused latent spaces (https://doi.org/10.1088/2632-2153/abe808). I wonder how merging two VAEs one for the ligands and another one for the proteins compares with the training strategies recommended in the present work.
Namely, I was thinking about the relation between the experience replay, that is somehow "forcing" the exploration of specific areas of the chemical space, and the optimization of the blended protein/ligand during the RL training procedure, that is inherently morphing the space to increase the density of high reward regions close to the point representing the protein of interest.
I would be interested in the opinion/point of view of the authors on this.

As reported, especially in the analysis presented in Figure 2, there is a trade-off between activity of the generated molecules and the SMILES validity. I wonder what would happen if the proposed strategies are adopted on models working with a more robust molecule representation, such as, SELFIES (https://doi.org/10.1088/2632-2153/aba947).
Leveraging the robustness of SELFIES strings can help to make the generation more efficient, in the sense that no regions of the chemical space would map to invalid decoded molecules, hence increasing the fraction of molecules considered. What are the thoughts on the authors on this?

On a final note, I want to tell the authors that I really enjoyed reading this paper, as a researcher in the field of accelerating molecular design with generative models, it is refreshing to read a piece of work that covers the aspect from data collection to experimental validation passing through model training so carefully written and with such well supported claims and results.

Reviewer #1 (Remarks to the Author):

This manuscript by Korshunova et al. reports heuristics for fine-tuning the performance of reinforcement-learning based molecular optimization approaches and the experimental validation of some computer-generated leads.

The work is interesting and the authors go well above and beyond by adding experimental validation to a very computational paper, which is rare and commendable. However, the manuscript suffers from this "split personality". The computational analysis is a relatively exhaustive exploration of model choices and hyperparameters. It is valuable and insightful but not intellectually surprising. To some degree, it is hard labor and clear performance metrics, but mostly journeyman in its vision.

Then the experimental validation really stands out among related papers, but is unfortunately not enough to carry the paper. The model predictions are fine but not groundbreaking (all kinase inhibitors look very much alike) and the models re-discovers chemistry that is not very surprising. Because custom synthesis is out of scope, the authors are forced to order from virtual catalogs, which they could have done by just screening the catalog with forward models.

Lastly, the language, the notation and the prose lack rigor some times.

In summary, i think this work may have all the building blocks for success, but they need to be better integrated and demonstrated.

Technical comments.

____

"Moreover, a naïve generative model pre-trained on a dataset of drug-like compounds such as ChEMBL23 would produce molecules with relatively high QED values" That may be the expectation - that a model retains the characteristics of the datasets it was trained on -, but is not necessarily the case and particularly not for synthetic accessibility in RL works. Is it possible to support this statement with literature references? Refs 10,20,24 report optimizing QED, not that high QED from the training data translated to the generated molecules.

Response:

The objective function used in training SMILES-based generative model is designed to match the distribution of a character given a prefix in generated samples to the one of training data. This will result in similar content of both individual atoms and fragments in molecules. This in turn will lead to the similar distribution of property values, assuming that the property values depend on which fragments molecules consist of.

We used this phenomenon during the transfer learning stage, which was initially introduced in Merk, D., Grisoni, F., Friedrich, L., & Schneider, G. (2018). Tuning artificial

intelligence on the de novo design of natural-product-inspired retinoid X receptor modulators. *Communications Chemistry*, *1*(1), 1-9.

Moreover, to illustrate this phenomenon we compared distribution of QED values from various sources of molecules – generated library, ChEMBL, Enamine REAL and DrugBank. The distributions were computed based on 10000 molecules randomly drawn from each subset and are shown in the figure below:

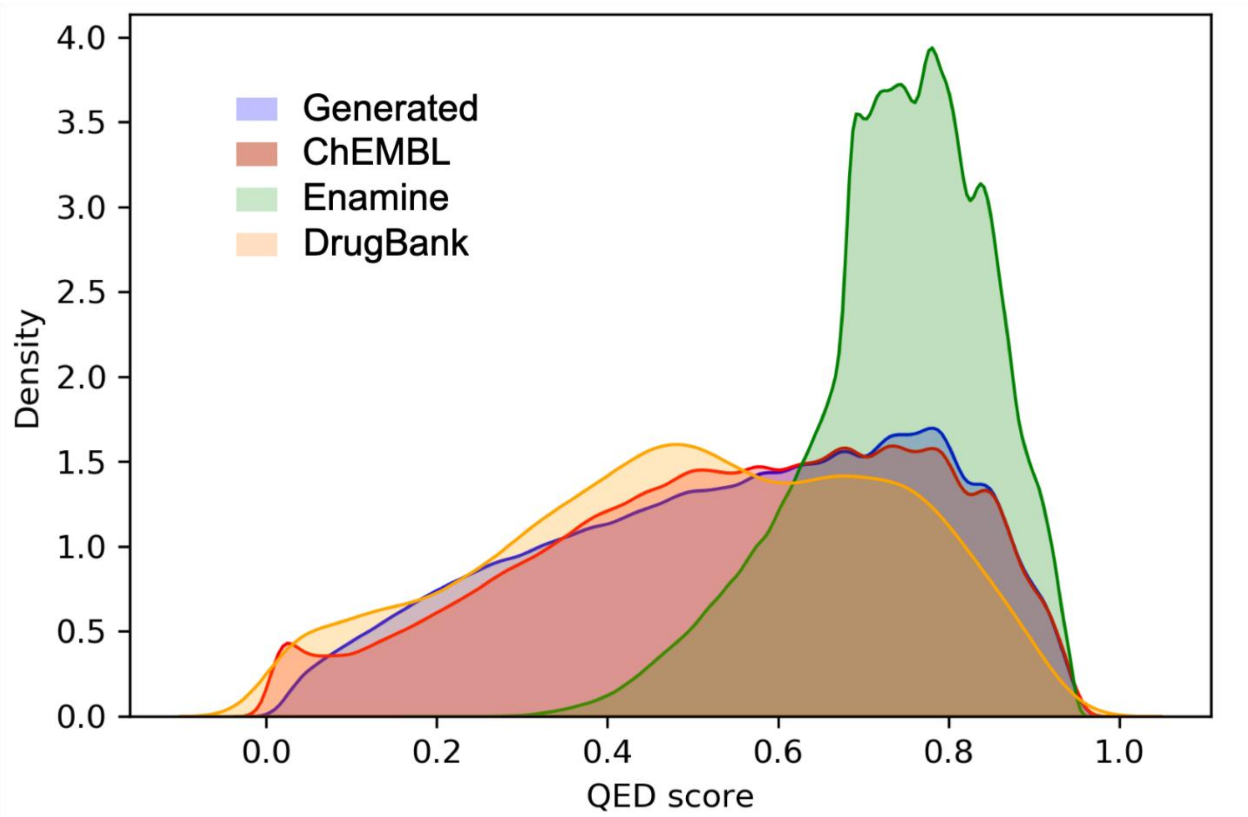

"In contrast to physical properties such as LogP that can be calculated directly from molecular structure" LogP is not calculated from structure through some physics law. It is predicted through fitted QSAR or ML model, much in the same way as a binding affinity is. The authors perhaps mean that the latter model has less training data than the former or that it's more non-linear.

Response:

In many computational papers on deep generative models people used Atom-based calculation of LogP Crippen's approach (https://www.rdkit.org/docs/source/rdkit.Chem.Crippen.html). For example:

- You, Jiaxuan, et al. "Graph convolutional policy network for goal-directed molecular graph generation." *arXiv preprint arXiv:1806.02473* (2018).

- Jin, Wengong, Regina Barzilay, and Tommi Jaakkola. "Junction tree variational autoencoder for molecular graph generation." *International conference on machine learning*. PMLR, 2018.

We clarified this point in the text, and corrected “LogP” to “cLogP”.

"However, unlike physical molecular properties like LogP that every molecule possesses, specific bioactivity is a target property that exists for only a small fraction of molecules, which leads to the reward sparseness in the generative models" This might be just poor phrasing, but all molecules will show binding affinity (most of them a very small one) towards any target. The statement is perhaps more applicable to classifiers, since mediocre and extremely bad binders all get the "negative" label. Still, a QSAR classifier will predict a continuous output. The discussion should disentangle sparseness _in the magnitude itself_ with sparsity in the availability of labeled data.

Response:
We agree that both regression and classification model will predict binding affinity for any molecule. However, for the vast majority of molecules, this binding affinity will be very low, which will result in low or zeros values of reward (not absence of reward). However, the lack of positive examples with higher values of reward will not provide enough feedback for the model to train.

None of the statements in line 82 to 91 are supported with literature references.

Response:
We apologize for this omission. Literature references were added appropriately.

"Training the generative network to optimize the potency of " Potency is a continuous variable that is in principle amenable to a continuous reward function. The previous comment stands: is this a data quantity problem? Or a problem of the nature of the measured variable?

Response:
There is a very low chance of observing a molecule with high potency when sampled randomly from the distribution of unoptimized generative model. During training procedure with RL, training examples are produced by the generative model. The model trained just on negative examples (molecules with low potency values) will unlikely discover positive examples (molecules with high potency values). We clarified this point the manuscript.

"could not discover any active molecules for EGFR due to sparse reward" How were molecules validated to be active?

Response:
Molecules were validated computationally with QSAR model. We apologize for confusing language. In the revised manuscript we consistently use “predicted active” or

“high predicted active class probability” for QSAR model evaluation and “active” or “bioactive” for experimental validation.

" generated molecules with high active class probabilities" I encourage the authors to use consistent language to refer to the outcomes of the approach. Is this equivalent to "active molecules" above?

Response:
We appreciate for pointing out for these inconsistencies. Indeed “generated molecules with high active class probabilities” and “active molecules” refer to the same concept in the context of computational experiment. We proofread the paper and did out best to fix inconsistencies in the language.

"As described above, we used the pre-trained ChEMBL model " Above where? The caption of Figure 1? "The model was pre-trained on ChEMBL data and then trained for 20 epochs." What is the difference between pre-train and train? It is not stated. Lines 149 - 155 repeat information in the caption.

Response:
Model training consists of 2 stages – pretraining the generator from scratch on a vast dataset such as ChEMBL in a supervised manner to produce mostly valid SMILES strings without any property optimization at this point. The second stage is training the model with RL to optimize the property values of the generated molecules. We added more clarification about this aspect into the manuscript.

"We assessed the extent of overfitting by recording the fraction of the generated trajectories that generate valid SMILES strings, which is defined as the ratio of valid and unique SMILES strings over the total number of the generated trajectories" Why is overfitting related to validity? Couldn't it also result in mode collapse?

Response:
The model can overfit with respect to the property predictor. For example, if the QSAR model assigns high potency to molecules with a specific chemical group, the generative model can discover and exploit it. Such scenario often leads to decrease in validity.
*We use ratio of **valid and unique** SMILES strings over the total number of generated trajectories. This metric would detect mode collapse, since we are discarding repeated molecules. We clarified this point in the revised manuscript.*

"with the arbitrary probability threshold of 0.75" A lot seems to be hinging on this choice. How is it justified? Does it modify the results if it changes? Which fraction of the activity test data is under this threshold?

Response:

This threshold reflects confidence of the QSAR model. We use ensemble of RFs as a predictor, and the final prediction is the mean of predictions from each model in the ensemble. Threshold of 0.75 ensures that majority of the models in the ensemble assigned high probability values to the input molecule. Changing this threshold is possible, however there is a trade-off between confidence in predictions and diversity of the molecules. Lower thresholds will result in more diverse subset with less reliable predictions.

"the valid fraction as a proxy for mode collapse. Two scenarios can decrease valid fraction: " Why not disentangle those two? Make one metric for unique strings and another for their validity? The text below suggests this partitioning is applicable.

Response:
We agree that disentangling validity and uniqueness rate is possible, however our metric also accurately represents both scenarios.

"as predicted by the random forest ensemble" What random forest ensemble? This hasn't been mentioned yet. The model should be introduced when it's first used. (line 165? or earlier)

Response:
We added description of the model to the text.

"remove molecules with Bemis Murcko scaffolds present in the historical EGFR data" This may be insufficient for heterocycles, where some C =>N or S (or vice versa) substitutions in a ring may switch scaffolds but not chemical similarity. I encourage the authors to replicate this with Tanimonoto-similarity spherical clustering instead of / in combination with the scaffold splits.

Response:
We agree that spherical clustering is a good way to accomplish the same goal and will consider using this approach in further work.

Figure 4 Caption. " The most common scaffolds had counts and percentages as follows" Why not just write the count under the scaffold as a number - percentage tuple?

Response:
This is a good suggestion to make this figure easier to read. We fixed the figure in the manuscript.

Figure 5. x axis say "predicted activity" In the same line as above, i encourage the authors to be more rigorous around what it is that the model is predicting. Is it a score, a probability, an activity ? Line 291-292 "progressively higher activities" The model does

not predict activity, it predicts the likelihood of being active. There is no reason to assume higher likelihood implies higher potency.

Response:
Thanks for pointing. We paid a close attention to the usage of word regarding potency/activity/probability and revised the text to make our language more consistent.

" With few notable exceptions, most of the current de novo design publications are purely computational" I wholeheartedly agree, and this is a tremendously strong point in favor of this work.

Response:
We greatly appreciate positive feedback from the Reviewer.

Ln 306 "high active class probabilities" The notation keeps changing.

Response:
Thanks for pointing. We paid attention to the usage of different notations referring to the same thing and improved it in the manuscript.

Ln 310 "synthesis of ultra-large chemical libraries" These libraries have not been synthesized. Enamine REAL is based on synthesizing arbitrary members of such virtual libraries.

Response:
We respectfully disagree, as this paper, ref 39 and other use cases show, Enamine shows over 80% success in synthesis of these virtual molecules. Indeed most of the compounds are virtual but they are readily available when needed.

Why are there only 4 molecules in Table 1 if 23 were ordered? It is important to show the full story. What other candidates were ordered but not active (maybe not the insoluble ones) ? How chemically dissimilar to the positives and to the training data were the negative controls? Are they truly a challenge or obvious inactive even to the eye. What about the 11 false positives? All this belongs in the main paper, IMO. What is meant by NN in the training set. Is this in the random forest training set or in the generative model training set?

Response:
Other purchased and tested candidates (including 5 negative controls and 11 false positives) are shown in the Supplementary Material Table S1. As a negative control, we selected five molecules predicted to be inactive but containing the same 4-anilinoquinazoline scaffold. We now expanded Table 1 with negative control compounds. By NN in the training set we mean dataset used to train Random Forest predictor model.

“Notably, the four active compounds contained only small substituents (Br, NH2, CH3) at the 4’ position of the 4-anilino group (Table S1) paired with halogen substitution on the 5, 6, or 8 positions of quinazoline core” Just like the NN in the training set?

Overall active molecules were quite diverse with respect to the R-group substituents.

Response:

"Surprisingly, however, the 4’-fluroanilino-6- fluroquinazoline analog was not active" All the more reason to bring these comparisons to the main text

This is a clear activity cliff, and we reflected this in revised text.

Response:

"Notably, all of the analogs with large linear or branched substituents at the 4' position were inactive in the enzyme assay" What about their NNs? Were they labeled as positive or negatives?

Unfortunately, their NN from the train data do not have meaningful analogs in this position. Nevertheless, we could speculate, that based on the crystal structure of with lapatinib inhibitor (3BBT), polar substituents at the 4' position might extend into a hydrophobic pocket, thus preventing Please see a revised text.

Response:

How would the approach compare in terms of computational cost with running an exhaustive search over Enamine REAL with the random forest model?

Response:
Although we did not explicitly benchmark our method to screening Enamine REAL, generative approach would likely be slightly faster since one need not to compute QSAR descriptors for the full Enamine REAL library. This computational advantage will only grow as ultra large libraries approaching explicitly enumerable sizes.

Minor language comments
"application of the deep generative recurrent neural network " This seems to be missing a word. The deep generated RNN _architecture_ ?

"enhanced by several novel technical tricks to designing experimentally validated potent inhibitors of the epidermal growth factor (EGFR)." This sentence is ambiguous - one cannot design experimentally validated compounds, typically one designs the compounds and then validates. The authors should clarify whether this is a prospective

or retrospective study.

"the success rate of finding novel bioactive compounds" Finding already implies it was a success

"molecule's SMILES"

"Problem of sparse rewards in reinforcement learning" missing the definite article.

"novel approach for discovering novel bioactive molecules"

"Neural network training is a nontrivial task as its hyperparameter values" hyperparameters of the task, the training or the network?

Lines 127-131 seem overly repetitive.

We sincere thank the Reviewer for their valuable comments and suggestions that helped us greatly improved the quality of the manuscript.

Reviewer #2 (Remarks to the Author):

The paper of M. Korshunova et al. reports a set of techniques - 1) fine-tuning, 2) experience replay, and 3) real-time reward shaping - applied in de novo design task named "a bag of tricks". As the authors mention, techniques 1 and 2 have been already proposed earlier, and their extension is demonstrated in the paper, whereas technique 3 is new. The authors conclude that the application of these techniques in combination with the reinforcement-learning approach improves the active molecules generation process for the given Neural Network architecture. The selected generated molecules were tested, and 2 compounds with an IC50 lower than 100 nM were discovered. This shows the importance of the study and the readiness of the proposed architecture to be used in a real drug discovery project. Still, some questions arise:

---

1) Is there a chance to do something similar for a graph-based model, and is there any sense to go there?

Response:
Trying similar approach with different types of generative model (including graph-based models) makes total sense. However, we leave it to the future work. We developed graph-based generative model in a separate publication (https://arxiv.org/abs/1905.13372) and we plan to do it as a follow-up study.

---

2) line 79 - it is better to replace "binary outputs" with "categorical outputs" to respect the multi-class classification tasks.

---

3) Figure 3 - Letters (A) and (B) are given in the caption but there is no such in the figure.

Response:
We sincere thank the Reviewer for their careful reading. We fixed this in the manuscript.

---

4) lines 418-420 - invisible symbols.
Response:
We fixed these and other typos in the manuscript.

---

5) lines 436-437 - The fine-tuning procedure was modified by moving from experimental to predicted values. Shouldn't this introduce some noise or "model's quality" bias to the results of the transfer learning?

Response:
Yes, since predicted values are just estimate of the ground truth affinity. However, we deliberately didn’t perform transfer learning on experimental data to avoid data leakage.

---

6) It is not clear whether compounds predicted as highly active are mixed with others in the experience replay or not?

Response:
Experience replay buffer only contained molecules with high rewards. We added this clarification to the revised manuscript.

---

7) line 249 - the meaning of the "non-zero actives" phrase is not clear. I assume it has the same meaning as "non-zero predicted probabilities" mentioned in lines 469-470, thus, it should be clarified.`

Response:
Correct, "non-zero actives" means "non-zero predicted probabilities". We clarified this in the manuscript.

Nevertheless, the paper is of good quality and it worths to be published.

We greatly appreciate positive feedback from the Reviewer.

Reviewer #3 (Remarks to the Author):

The authors present a study on the application of reinforcement learning and generative modeling to the use case of designing active inhibitors against EGFR, a key protein in various cancer types.
The work presents in a clear fashion a series of "tricks" and techniques that can be exploited to improve known issues of molecular design, especially the sparse reward dilemma, that usually hinders the ability to explore relevant regions of the molecular space, hence limiting the capacity to generate/design consistently active candidates.

I believe that the paper can be considered ready for publication even in the current form, but here I leave some comments that I think could help in expanding the discussion and increase the, already high, quality of the work.

Always within the realm of RL applications to molecular design, another recent work alleviated the sparsity of the reward by considering a multi-modal predictor that acts on fused latent spaces (https://doi.org/10.1088/2632-2153/abe808). I wonder how merging two VAEs one for the ligands and another one for the proteins compares with the training strategies recommended in the present work.

Response:
To properly answer this question one should perform extensive benchmarking of 2 approaches. However, heuristics we propose are model agnostic as long as the task can be formulated as a reinforcement learning problem, and can be combined with multi-model predictor on fused latent spaces proposed in the mentioned work.
We thank the Reviewer for mentioning this relevant work, we cited it in our manuscript.

Namely, I was thinking about the relation between the experience replay, that is somehow "forcing" the exploration of specific areas of the chemical space, and the optimization of the blended protein/ligand during the RL training procedure, that is inherently morphing the space to increase the density of high reward regions close to the point representing the protein of interest.
I would be interested in the opinion/point of view of the authors on this.

Response:
We do not explicitly consider information about target protein in our work. However, if one can build a predictor that uses this information (molecular docking, for example), our heuristics will still be applicable.

As reported, especially in the analysis presented in Figure 2, there is a trade-off between activity of the generated molecules and the SMILES validity. I wonder what would happen if the proposed strategies are adopted on models working with a more

robust molecule representation, such as, SELFIES (https://doi.org/10.1088/2632-2153/aba947).
Leveraging the robustness of SELFIES strings can help to make the generation more efficient, in the sense that no regions of the chemical space would map to invalid decoded molecules, hence increasing the fraction of molecules considered. What are the thoughts on the authors on this?

Response:
We agree that it is absolutely worth exploring the effect of using SELFIES as representation. However, we leave it to the future work.

On a final note, I want to tell the authors that I really enjoyed reading this paper, as a researcher in the field of accelerating molecular design with generative models, it is refreshing to read a piece of work that covers the aspect from data collection to experimental validation passing through model training so carefully written and with such well supported claims and results.

We sincerely thank the Reviewer for insightful comments and high evaluation of our work.

REVIEWERS' COMMENTS:

Reviewer #1 (Remarks to the Author):

The authors have successfully addressed all the comments and, i insist, gone above and beyond with experimental validation of computational predictions.
